# Abnormal RNA stability in amyotrophic lateral sclerosis

**DOI:** 10.1038/s41467-018-05049-z

**Published:** 2018-07-20

**Authors:** E. M. Tank, C. Figueroa-Romero, L. M. Hinder, K. Bedi, H. C. Archbold, X. Li, K. Weskamp, N. Safren, X. Paez-Colasante, C. Pacut, S. Thumma, M. T. Paulsen, K. Guo, J. Hur, M. Ljungman, E. L. Feldman, S. J. Barmada

**Affiliations:** 1 Department of Neurology, University of Michigan Medical School, Ann Arbor, MI 48109 USA; 2 Department of Radiation Oncology, University of Michigan Medical School, Ann Arbor, MI 48109 USA; 3 Department of Biomedical Sciences, School of Medicine and Health Sciences, University of North Dakota, Grand Forks, ND 58202 USA; 4 Cellular & Molecular Biology Program, University of Michigan Medical School, Ann Arbor, MI 48109 USA; 5 Neuroscience Graduate Program, University of Michigan Medical School, Ann Arbor, MI 48109 USA

## Abstract

Amyotrophic lateral sclerosis (ALS) and frontotemporal dementia (FTD) share key features, including accumulation of the RNA-binding protein TDP-43. TDP-43 regulates RNA homeostasis, but it remains unclear whether RNA stability is affected in these disorders. We use Bru-seq and BruChase-seq to assess genome-wide RNA stability in ALS patient-derived cells, demonstrating profound destabilization of ribosomal and mitochondrial transcripts. This pattern is recapitulated by TDP-43 overexpression, suggesting a primary role for TDP-43 in RNA destabilization, and in postmortem samples from ALS and FTD patients. Proteomics and functional studies illustrate corresponding reductions in mitochondrial components and compensatory increases in protein synthesis. Collectively, these observations suggest that TDP-43 deposition leads to targeted RNA instability in ALS and FTD, and may ultimately cause cell death by disrupting energy production and protein synthesis pathways.

## Abstract (web summary)

RNA instability may be a contributing factor in amyotrophic lateral sclerosis (ALS). Here the authors carry out genome-wide assessment of RNA synthesis and stability in cells derived from patients with C9orf72 ALS, sporadic ALS and healthy controls.

## Introduction

In amyotrophic lateral sclerosis (ALS), progressive degeneration of motor neurons leads to muscle atrophy and weakness^1^. Additionally, nearly half of ALS patients show cognitive changes akin to frontotemporal dementia (FTD), including impulsive behavior and language deficits^2^. Supporting the connection between ALS and FTD, affected neurons and glia in both disorders accumulate TDP-43 (transactive response DNA/RNA-binding protein 43 kDa)^3^. This accumulation is characteristic of sporadic as well as familial disease, including that caused by hexanucleotide *C9orf72* (chromosome 9 open reading frame 72) expansions, the most prevalent mutation underlying both ALS and FTD^4,5^. Together, these findings support a pathological role for TDP-43 deposition in these disorders^6,7^.

Mounting evidence shows that abnormal RNA homeostasis underlies neurodegeneration in the majority of those with ALS and FTD^8,9^. Mutations in the genes encoding several RNA-binding proteins (RBPs) result in ALS, FTD, and related disorders^8^. Approximately 1/3 of all transcribed RNAs harbor TDP-43 binding sites, consistent with the critical functions of TDP-43 in regulating RNA splicing and transport^10–12^. Genetic ablation of TDP-43 is lethal^13^, while its overexpression triggers cell death in multiple models, from yeast to nonhuman primates^14–17^. TDP-43 knockdown results in abnormal splicing and the inclusion of unannotated exons within hundreds of transcripts^18,19^; in most cases, the introduction of such “cryptic exons” changes the coding sequence or shifts the reading frame, either of which could result in the introduction of premature termination codons and nonsense-mediated RNA decay (NMD)^20^. Additionally, overexpression of an essential NMD component, UPF1 (up frameshift 1), enhances RNA decay and rescues neurodegeneration resulting from TDP-43 overexpression^21,22^. These observations emphasize the crucial connection between RNA stability and neurodegeneration in ALS and FTD.

RNA degradation is a tightly regulated process required for RNA homeostasis and, by extension, protein expression^23^. While prior investigations uncovered substantive abnormalities in RNA abundance in disease models and in ALS/FTD patient tissues^10,11^, it remains unclear whether such differences are due to dysfunctional synthesis or degradation of RNA transcripts. In part, the contribution of RNA degradation to ALS and FTD has remained obscure because of the lack of available methods for studying the pathways and molecules involved. Traditional techniques for investigating RNA stability require the use of transcriptional inhibitors that have adverse effects on cellular health and RNA processing. To overcome these limitations, we use Bru-seq and BruChase-seq^24^, innovative methods that enable genome-wide assessment of RNA synthesis and stability in living cells. Fibroblasts and human-induced pluripotent stem cells (iPSCs) derived from individuals with sporadic and familial ALS due to *C9orf72* mutations display consistent abnormalities in the stability of RNA transcripts encoding components of two pathways essential for cellular function and survival—oxidative phosphorylation and protein synthesis. These patterns of RNA instability are conserved in postmortem samples, and are mirrored in proteomic and functional studies of patient-derived samples. Taken together, these studies suggest that a basic failure of RNA homeostasis in ALS and FTD can, over time, affect energy production and protein translation, eventually resulting in cell death.

## Results

### RNA destabilization in ALS patient-derived fibroblasts

**Fig. 1:**
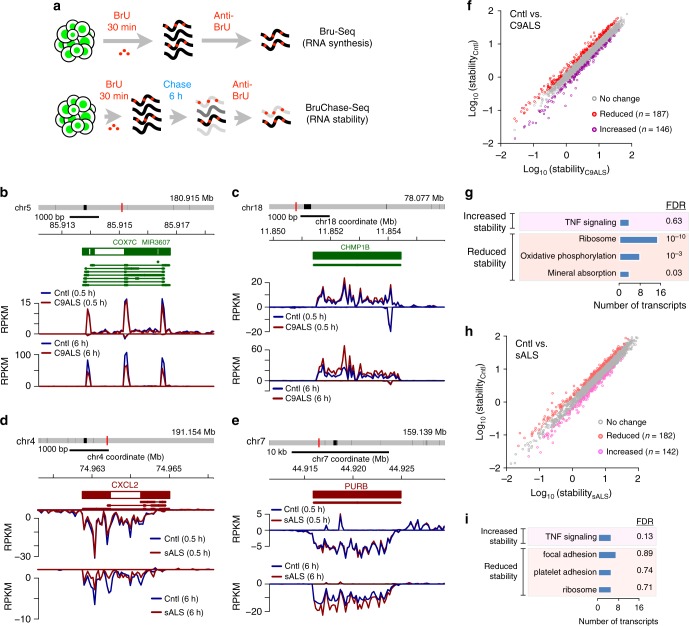
RNA destabilization in ALS fibroblasts. **a** Schematic of Bru-seq (top) and BruChase-seq (bottom). BrU Bromouridine, Anti-BrU antibodies that recognize BrU. Example traces of RNA transcripts destabilized (**b**) or stabilized (**c**) in C9ALS fibroblasts. Blocks and lines denote gene and transcript structure, respectively. + strand genes are in green, – strand genes are in red. The traces represent RNA abundance at 0.5 h (top) and at 6 h (bottom) following Bru labeling. RPKM reads/kilobase of transcript/million mapped reads. Representative examples of RNA transcripts destabilized (**d**) or stabilized (**e**) in sALS fibroblasts. Scatter plot (**f**) and gene ontology (**g**) for 333 transcripts showing a change in stability ≥1.5-fold in C9ALS fibroblasts, in comparison to control (Cntl) cells. Stability is calculated as a ratio of transcript abundance at 6 h vs. 0.5 h. Scatter plot (**h**) and gene ontology (**i**) for 324 transcripts showing a change in stability ≥1.5-fold in sALS fibroblasts, compared to control cells. FDR false discovery rate. Cell lines used for these experiments are listed in Supplementary Table 1

We asked if global or specific alterations in RNA stability are characteristic of ALS by applying Bru-seq and BruChase-seq^24^ to 14 fibroblast cell lines obtained from individuals with *C9orf72*-linked familial ALS (C9ALS, four lines), sporadic ALS (sALS, five lines), or controls (five lines; Supplementary Table 1). These methods capture newly synthesized RNA by metabolic labeling of nascent RNA transcripts with bromouridine (BrU), followed by immunoprecipitation of BrU-labeled RNA and deep sequencing (Fig. 1a). The stability of each transcript was assessed by comparing the corresponding reads after 0.5 h of labeling (Bru-seq) to that after a 6 h chase in uridine (BruChase-seq). Out of 22,977 annotated transcripts, we identified 333 RNAs whose stability was altered ≥1.5-fold in C9ALS fibroblasts (Fig. 1b–e; Supplementary Data 1), 56% of which were destabilized (Fig. 1f). Gene set enrichment analysis using gene ontology (GO)^25^ revealed that the ribosome and oxidative phosphorylation pathways were highly enriched among destabilized transcripts (false discovery rate (FDR) < 0.05; Fig. 1g), while no pathways were enriched among stabilized RNAs.

In sALS fibroblasts, 324 transcripts demonstrated a change in stability ≥1.5-fold compared to controls (Fig. 1h; Supplementary Data 1). For a subset of transcripts tested, changes in RNA stabilization were confirmed by quantitative RT-PCR (qPCR) (Supplementary Fig. 1a). Few pathways were enriched among stabilized or destabilized RNAs in sALS fibroblasts (Fig. 1i). Approximately 1/3 of stabilized transcripts and 1/5 of destabilized transcripts were common to sALS and C9ALS fibroblasts (Supplementary Fig. 1b, c). Even so, no pathways were enriched among the overlapping transcripts, arguing against conserved patterns of altered RNA stability in C9ALS and sALS fibroblasts.

**Fig. 2:**
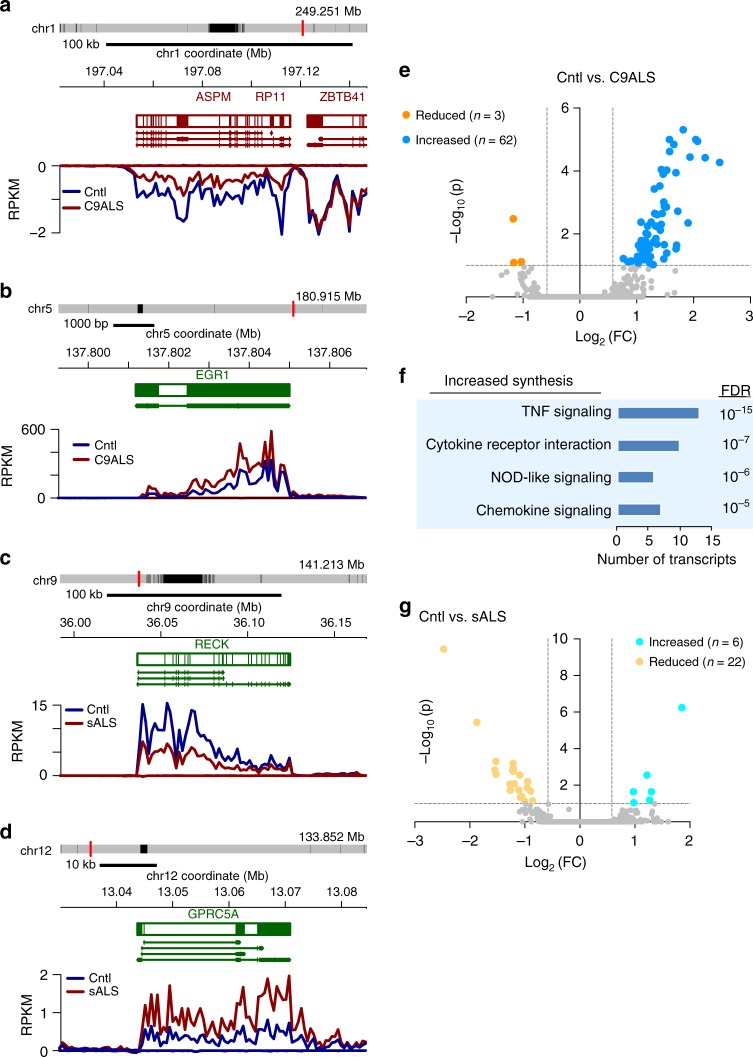
Abnormal RNA synthesis in ALS fibroblasts. Examples of RNA transcripts showing reduced (**a**) and increased (**b**) synthesis in C9ALS fibroblasts. The traces represent RNA abundance 0.5 h following Bru labeling. Representative schematics of RNA transcripts exhibiting reduced (**c**) and increased (**d**) synthesis in sALS fibroblasts. **e** Volcano plot for 65 transcripts showing significant changes in synthesis in C9ALS fibroblasts, in comparison to control (Cntl) cells. **f** Gene ontology was performed for transcripts displaying reduced synthesis in C9ALS fibroblasts. FDR false discovery rate. **g** Volcano plot for 28 transcripts exhibiting significant changes in synthesis in sALS fibroblasts, compared to control cells. Dotted lines in **e** and **g** depict an adjusted *p* value of 1 and fold change (FC) of 1.5. Cell lines used for these experiments are listed in Supplementary Table 1

From the Bru-seq data, we identified multiple transcripts whose synthesis differed ≥1.5-fold in controls vs. C9ALS or sALS fibroblasts (Fig. 2a–d; Supplementary Data 2). Nearly all (95%) of the 65 transcripts exhibiting altered synthesis in C9ALS fibroblasts displayed increased rates of production (Fig. 2e)—these RNAs were significantly enriched for inflammatory signaling pathways by GO analysis (Fig. 2f). Only 6 transcripts exhibited increased production in sALS fibroblasts compared to controls, while 22 displayed reduced synthesis (Fig. 2g). No pathways were significantly enriched in either group by GO. Only two transcripts showed increased synthesis in both C9ALS and sALS fibroblasts (MMP1 and NR4A2), while one transcript exhibited a common reduction in synthesis (DACT1; Supplementary Fig. 2 and Supplementary Table 2).

### A conserved pattern of RNA destabilization in ALS iPSCs

Given the substantial heterogeneity of fibroblasts in culture^26^, we were concerned that relevant differences between C9ALS, sALS, and control cells could be obscured. We also questioned whether the observed changes in RNA synthesis and stability would be maintained in cell types other than fibroblasts. Therefore, we reprogrammed a subset of fibroblasts (Supplementary Table 1) into iPSCs using integration-free approaches^27,28^, verified their pluripotency (Supplementary Fig. 3), and assessed the production and turnover of RNA transcripts by Bru-seq and BruChase-seq.

**Fig. 3:**
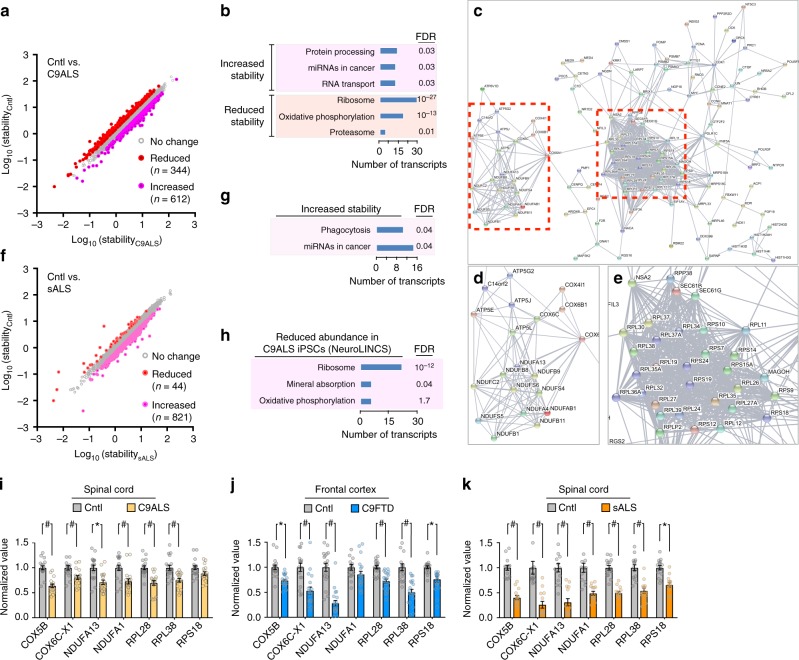
Conserved patterns of RNA destabilization in C9ALS iPSCs. Scatter plot (**a**), gene ontology (**b**), and STRING analysis (**c**) for the 956 transcripts showing changes in stability ≥1.5-fold in C9ALS iPSCs, in comparison to control (Cntl) cells. Higher resolution views for boxed areas in **c** are shown in **d** and **e**. Scatter plot (**f**) and gene ontology (**g**) for 865 transcripts with altered stability ≥1.5-fold in sALS iPSCs, in comparison to control cells. **h** Gene ontology analysis of RNA transcripts showing ≥1.5-fold reduction in C9ALS iPSCs vs. controls, acquired from the NeuroLINCS database. FDR false discovery rate. Cell lines used for these experiments, performed in duplicate, are listed in Supplementary Table 1. **i−k** Quantitative (q)RT-PCR in patient postmortem tissue, showing reduced abundance of RNAs related to mitochondrial oxidative phosphorylation (COX6B, COX6C-X1, COX5B, NDUFA1, NDUFA13) and the ribosome (RPL28, RPL38, RPS18) in C9ALS spinal cord (**i**), C9FTD frontal cortex (**j**), and sALS spinal cord (**k**). Graphs in **i−k** depict mean ± standard error. *p < 0.05, #*p* < 0.01, two-way ANOVA with Sidak’s multiple comparison test. All data in **i−k** were assembled from ≥3 biological replicates. Supplementary Table 3 lists the number of samples in each condition and clinical characteristics for each patient

From 22,984 annotated transcripts, we identified several hundred demonstrating a change in stability ≥1.5-fold over controls in ALS iPSCs (Supplementary Fig. 4a−d; Supplementary Data 3). In C9ALS iPSCs, 956 transcripts demonstrated differences in stability (Fig. 3a), with 36% destabilized ≥1.5-fold. For a subset of transcripts, changes in RNA stability were verified by qRT-PCR (Supplementary Fig. 5a). GO analysis highlighted a profound enrichment in ribosomal and oxidative phosphorylation pathways among destabilized transcripts in C9ALS iPSCs (Fig. 3b), reinforcing the results from C9ALS fibroblasts (Fig. 1g). Network analysis using STRING, a method for illustrating interactions among genes and proteins^25^, emphasized the strong enrichment for ribosomal and mitochondrial processes in this dataset (Fig. 3c–e).

Nearly all (95%) of the 865 transcripts demonstrating ≥1.5-fold change in sALS iPSCs were stabilized (Fig. 3f; Supplementary Data 3). These were modestly enriched in phagocytic and miRNA pathways, as determined by GO analysis (Fig. 3g); the latter pathway was also enriched in transcripts stabilized in C9ALS iPSCs (compared to Fig. 3b). No pathways were significantly enriched among the 44 destabilized RNAs in sALS iPSCs. Venn diagrams highlighted the 30−40% overlap of stabilized RNA transcripts in C9ALS and sALS iPSCs (Supplementary Fig. 5b), but no pathways were enriched among these transcripts by GO. Only 17 RNAs were commonly destabilized in both groups (Supplementary Fig. 5c), showing no enrichment for specific pathways.

To validate these results and explore their consistency across distinct datasets, we compared RNA abundance in C9ALS and control iPSCs using publicly available RNA-seq data generated by the NeuroLINCS consortium^29^. Transcripts downregulated ≥1.5-fold in C9ALS iPSCs were highly enriched for the ribosome pathway and mildly enriched for the mineral absorption pathway by GO analysis (Fig. 3h). We also detected a trend towards enrichment for the oxidative phosphorylation pathway, indicating consistent dysregulation of ribosomal and oxidative phosphorylation transcripts in C9ALS iPSCs.

To determine if this pattern was conserved in the human central nervous system (CNS), we examined select transcripts in postmortem cortex and spinal cord from ALS and FTD patients using qRT-PCR (Supplementary Table 3). In spinal cord from C9ALS patients, and in frontal cortex from *C9orf72*-mutant FTD patients (C9FTD), we detected a significant reduction of ribosome protein-encoding RNAs and oxidative phosphorylation RNAs (Fig. 3i, j). sALS spinal cord exhibited analogous changes (Fig. 3k), suggesting that the distinct patterns of RNA destabilization observed in fibroblasts and iPSCs are reflected in the CNS of human ALS and FTD patients.

In addition to RNA stability, we also investigated RNA synthesis in C9ALS and sALS iPSCs by Bru-seq (Supplementary Fig. 6a−d; Supplementary Data 4). We observed 834 transcripts showing a change in synthesis ≥1.5-fold over controls in C9ALS iPSCs (Supplementary Fig. 6e). Pathways involving focal adhesion and actin cytoskeleton were significantly enriched among RNAs displaying increased synthesis, while transcripts implicated in oxidative phosphorylation and Parkinson’s disease were enriched among RNAs exhibiting reduced synthesis in C9ALS iPSCs (Supplementary Fig. 6f). Among the 541 RNAs exhibiting altered synthesis in sALS iPSCs (Supplementary Fig. 6g), only those involved in rap1 signaling were significantly enriched (Supplementary Fig. 6h). Despite the substantial overlap of transcripts showing increased synthesis in C9ALS and sALS iPSCs (Supplementary Fig. 7a), we observed only moderate enrichment for the focal adhesion pathway. As with fibroblasts, no pathways were enriched among transcripts exhibiting reduced synthesis in C9ALS and sALS iPSCs (Supplementary Fig. 7b).

### Transcript characteristics associated with RNA stability

In fibroblasts and iPSCs, transcript length was proportional to stability—shorter transcripts were unstable, while longer transcripts were more stable (Supplementary Fig. 8a, b). Similar relationships were observed for 3′UTR length, which is independently associated with susceptibility to NMD and RNA interference^30^ (Supplementary Fig. 8c, d). Transcripts exhibiting a change in stability, either increased or decreased, exhibited fewer introns in C9ALS and sALS fibroblasts; although an analogous trend was noted in iPSCs, the effect was less clear (Supplementary Fig. 8e, f).

RBPs regulate RNA stability by shuttling transcripts to processing bodies for degradation, or sequestration and stabilization of transcripts within cytoplasmic stress granules^31,32^. We therefore asked whether the relative abundance of specific RBP recognition sites among affected transcripts might drive altered RNA stability in ALS patient-derived cells. Motifs recognized by a series of RBPs that form stress granules were enriched among the 3′UTRs of transcripts exhibiting altered stability in ALS iPSCs (Supplementary Fig. 9). Moreover, many of the corresponding RBPs, including TIA1, FUS, and TDP-43, are genetically or functionally linked with ALS, highlighting their implicit role in disease pathogenesis^8^.

### TDP-43 expression mimics RNA instability in control iPSCs

**Fig. 4:**
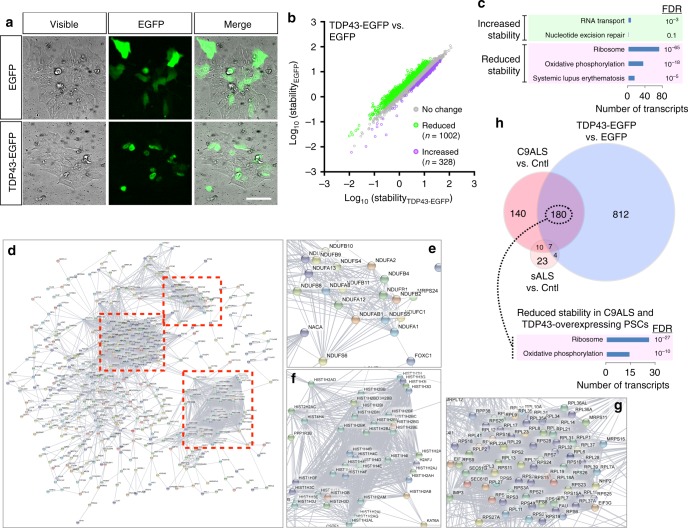
TDP-43 overexpression recapitulates RNA instability in control iPSCs. **a** Exogenous overexpression of EGFP and TDP-43 fused to EGFP (TDP43-EGFP) in control (Cntl) iPSCs. Scale bar, 100 µm. **b** Scatter plot for 1330 transcripts showing altered stability ≥1.5-fold in TDP43-EGFP overexpressing iPSCs, in comparison to EGFP-expressing cells, performed in triplicate. Gene ontology (**c**) and STRING analysis (**d**, excluding ubiquitin) for transcripts demonstrating altered stability in TDP43-EGFP overexpressing iPSCs. **e**−**g** Higher resolution views corresponding to boxed areas in **d**. **h** 180 transcripts were commonly destabilized in C9ALS and TDP43-EGFP-expressing cells. Among these transcripts, the ribosome and oxidative phosphorylation pathways were highly enriched by gene ontology. FDR false discovery rate

Given the enrichment for TDP-43 motifs among transcripts showing altered stability in ALS iPSCs (Supplementary Fig. 9), the critical function of TDP-43 in RNA splicing^10,11,18^, and the central connections between TDP-43 deposition and neurodegeneration in both sALS and C9ALS^3,5^, we surmised that TDP-43 accumulation could contribute to RNA instability in ALS patient cell lines. To test this, we overexpressed TDP-43 fused to enhanced green fluorescent protein (TDP43-EGFP) or EGFP itself in control iPSCs (Fig. 4a), and compared RNA stability in the two groups by BruChase-seq. TDP43-EGFP overexpression induced profound RNA destabilization—of the 1330 transcripts displaying ≥1.5-fold change in stability, 75% were destabilized (Fig. 4b; Supplementary Data 3). As in C9ALS fibroblasts and iPSCs, the ribosomal and oxidative phosphorylation pathways were highly enriched among TDP43-EGFP destabilized transcripts by GO (Fig. 4c). Network analysis confirmed this distinct pattern of RNA destabilization (Fig. 4d–g), outlining well-demarcated clusters centering on ribosomal, mitochondrial, and nucleosomal pathways. GO analysis also highlighted moderate enrichment for RNA transport and nucleotide excision repair pathways among stabilized transcripts (Fig. 4c). Previous studies link both pathways to ALS^33,34^, testifying to the relevance of TDP-43 and its downstream targets for disease pathogenesis.

One hundred and eighty transcripts were commonly destabilized in C9ALS and TDP43-EGFP-expressing iPSCs (Fig. 4h), accounting for 56% of RNAs destabilized in C9ALS iPSCs. Among these, the ribosome and oxidative phosphorylation pathways were highly and significantly enriched by GO. In the set of commonly destabilized RNAs in C9ALS and TDP43-EGFP overexpressing iPSCs (Supplementary Fig. 10), STRING illustrated clear clusters within the ribosomal and oxidative phosphorylation pathways. These data provide strong support that TDP43-EGFP expression is sufficient to produce a characteristic pattern of RNA instability—one also observed in C9ALS fibroblasts and iPSCs—involving the prominent destabilization of ribosomal and oxidative phosphorylation transcripts. Remarkably, TDP43-EGFP expression had virtually no effect on RNA synthesis as determined by Bru-seq (Supplementary Fig. 11; Supplementary Data 4), suggesting a relatively selective role for TDP-43 in post-transcriptional regulation of gene expression.

### Consequences of RNA instability for proteins in iPSCs

**Fig. 5:**
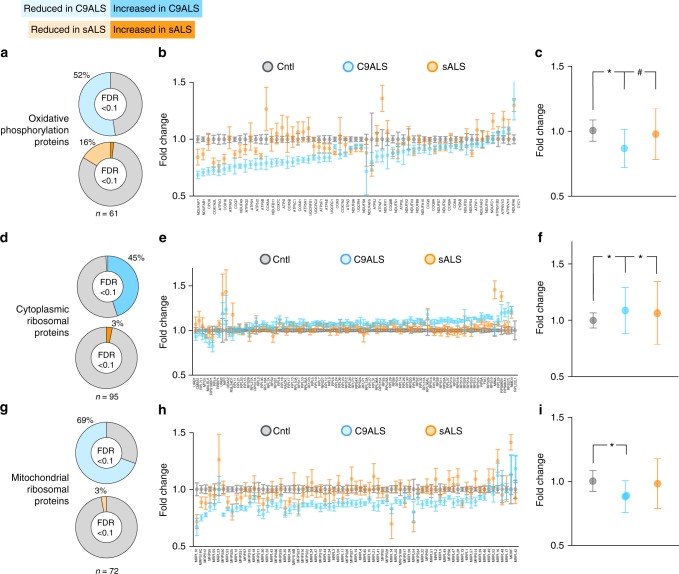
Reduced abundance of mitochondrial proteins in C9ALS iPSCs. **a** Of the oxidative phosphorylation proteins detected by MS (*n* = 61), 52% were significantly reduced in C9ALS iPSCs, and 16% were similarly reduced in sALS iPSCs compared to controls (Cntl). **b** Fold change in each of 61 oxidative phosphorylation proteins. **c** Cumulative change in the abundance of oxidative phosphorylation proteins in ALS iPSCs. **d** 45% and 3% of the 95 cytoplasmic ribosomal proteins detected by MS in ALS iPSCs were significantly increased in C9ALS and sALS iPSCs, respectively, compared to controls. **e** Fold change for the 95 cytoplasmic ribosomal proteins in ALS iPSCs. **f** Cumulative change in the abundance of cytoplasmic ribosomal proteins in ALS iPSCs. **g** 72 separate mitochondrial ribosomal proteins were detected by MS in ALS iPSCs; 69% and 3% were reduced in abundance in C9ALS and sALS iPSCs, respectively. **h** Fold change for the 72 mitochondrial ribosomal proteins in ALS iPSCs. **i** Cumulative change in the abundance of mitochondrial ribosomal proteins in ALS iPSCs. FDR false discovery rate. All experiments performed in triplicate, with two lines /condition. Plots in **c**, **f**, **i** show mean ± standard deviation. *p < 0.0001; #*p* < 0.05, one-way ANOVA with Benjamini−Hochberg correction for multiple observations

To assess the impact of RNA destabilization for ribosomal and oxidative phosphorylation proteins, we took advantage of tandem mass spectroscopy (MS) and simultaneously assessed all measurable components of these pathways in control, C9ALS and sALS iPSCs (Fig. 5; Supplementary Data 5). A total of 61 oxidative phosphorylation proteins were detected in iPSCs by MS. Of these, 52% were significantly reduced in C9ALS iPSCs, and 16% were reduced in sALS iPSCs (Fig. 5a–c). Concordantly, the cumulative abundance of oxidative phosphorylation components was significantly reduced in C9ALS iPSCs, and less so in sALS iPSCs, in comparison to controls. These data are consistent with the destabilization of oxidative phosphorylation transcripts in C9ALS fibroblasts and iPSCs (Figs. 1, 3), and further suggest that oxidative phosphorylation abnormalities may exist in sALS iPSCs independent of RNA instability.

We next asked if ribosomal proteins are likewise affected in ALS iPSCs. For these studies, we separated ribosome-related proteins into two groups: cytoplasmic and mitochondrial ribosomal proteins. Contrary to our expectations based on the observed destabilization of ribosomal protein-encoding RNAs in C9ALS cells, 45% of the 95 cytoplasmic ribosome proteins identified by MS exhibited a significant but subtle increase in abundance in C9ALS iPSCs (Fig. 5d–f), a finding confirmed for a subset of proteins by immunoblotting (Supplementary Fig. 12). Only 3% showed a similar increase in sALS iPSCs, compared to controls. When the abundance of all cytoplasmic ribosomal proteins was measured in aggregate, both C9ALS and sALS iPSCs demonstrated significant but modest increases in comparison to controls. In contrast, C9ALS iPSCs demonstrated a selective reduction in the abundance of mitochondrial ribosomal proteins (Fig. 5g–i). Of the 72 mitochondrial ribosomal proteins detected by MS, 69% were significantly reduced in C9ALS iPSCs, and 3% were reduced in sALS iPSCs, compared to controls.

**Fig. 6:**
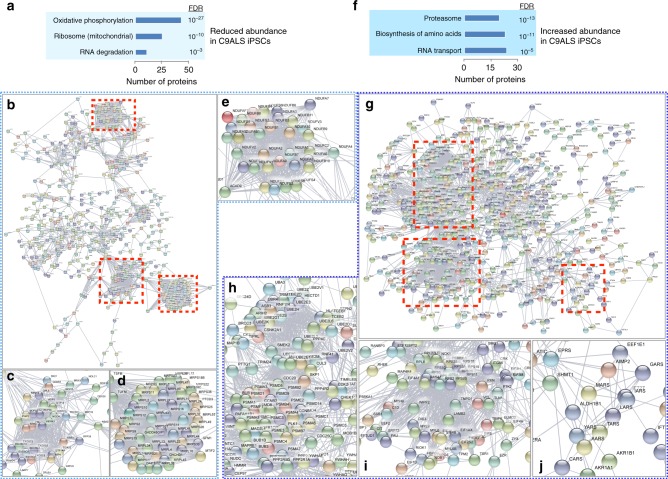
Unbiased proteomics confirms mitochondrial protein deficit in C9ALS iPSCs. Gene ontology (**a**) and STRING analysis (**b**) for the 806 proteins reduced ≥10% in C9ALS iPSCs, demonstrating enrichment for components of oxidative phosphorylation, mitochondrial ribosome, and RNA degradation pathways. Higher magnification views of boxed clusters in **b** are shown in **c−e**. Gene ontology (**f**) and STRING analysis (**g**) for the 961 proteins increased ≥10% in C9ALS iPSCs, showing enrichment for components of the proteasome, amino acid biosynthesis, and RNA transport pathways. Higher magnification views of boxed clusters in **g** are shown in **h−j**. FDR false discovery rate. All experiments performed in triplicate with two lines/condition

We also conducted an unbiased overview of all proteins showing a significant change in C9ALS and sALS iPSCs vs. controls. We identified 806 proteins that were significantly and consistently reduced ≥10% in C9ALS iPSCs (Fig. 6a). GO and network analysis using STRING highlighted oxidative phosphorylation constituents and mitochondrial ribosomal subunits among this set of proteins (Fig. 6b–e), confirming the results of investigations that focused specifically on these pathways (Fig. 5). We also noted a significant enrichment for components of the RNA exosome^35^ among proteins that were significantly reduced in C9ALS iPSCs, suggesting abnormal RNA decay machinery in these cells.

Because previous studies noted direct associations between mitochondrial ribosomes and glycine-arginine (GR) dipeptide repeat proteins encoded by the *C9orf72* repeat expansion^36^, we wondered whether the observed changes in mitochondrial proteins might be related to mitochondrial GR deposition in C9ALS iPSCs. However, we detected no accumulation of GR dipeptides within mitochondria of C9ALS iPSCs by immunocytochemistry (Supplementary Fig. 13), suggesting that the reductions in mitochondrial proteins in C9ALS iPSCs are independent of direct binding by GR. These findings are also consistent with the destabilization of ribosomal and oxidative phosphorylation RNAs in TDP43-overexpressing iPSCs (Fig. 4) that lack GR dipeptides.

The mild but significant upregulation of cytoplasmic ribosomal proteins in C9ALS iPSCs (Fig. 5b) contrasted with the relative instability of ribosome protein-encoding RNAs noted in these cells (Figs. 1, 3). We therefore asked if the observed increase in cytoplasmic ribosome proteins might represent a compensatory change intended to preserve the cell’s capacity to synthesize proteins. In support of this hypothesis, we detected significant upregulation of the protein biosynthesis machinery in C9ALS iPSCs (Fig. 6f). GO and STRING network analysis confirmed the relative enrichment of the amino acid synthesis pathway and further highlighted the proteasomal and RNA transport pathways (Fig. 6g–j). These data suggest that reductions in the stability of ribosome protein-encoding transcripts may be balanced in C9ALS iPSCs by upregulation of ribosomes and elements of the protein synthesis pathway.

We next asked if similar pathways are affected in sALS iPSCs. Unbiased assessment of proteins downregulated ≥10% in sALS iPSCs demonstrated enrichment for components of the oxidative phosphorylation pathway and tricarboxylic acid (TCA) cycle (Supplementary Fig. 14a). Of the 316 proteins exhibiting reduced abundance in sALS iPSCs, 75% were also downregulated in C9ALS iPSCs (Supplementary Fig. 14b). Within this conserved set, GO analysis highlighted both oxidative phosphorylation and the TCA cycle, indicative of shared deficiencies in energy production machinery in C9ALS and sALS patient-derived cells (Supplementary Fig. 14c). We also detected significant upregulation of proteins involved in ubiquitin-mediated proteolysis and amino acid biosynthesis in sALS iPSCs (Supplementary Fig. 14d), similar to what we observed in C9ALS iPSCs (Fig. 6). In fact, 68% of the proteins increased ≥10% in sALS iPSCs were also upregulated in C9ALS iPSCs (Supplementary Fig. 14e). Both amino acid synthesis and ubiquitin-mediated proteolysis pathways were significantly enriched among commonly upregulated proteins in C9ALS and sALS (Supplementary Fig. 14f). Taken together, these data reveal conserved downregulation of energy production pathways coincident with upregulation of protein synthesis and ubiquitin-mediated proteolytic pathways in C9ALS and sALS iPSCs.

### Correlating RNA stability and protein abundance in ALS iPSCs

**Fig. 7:**
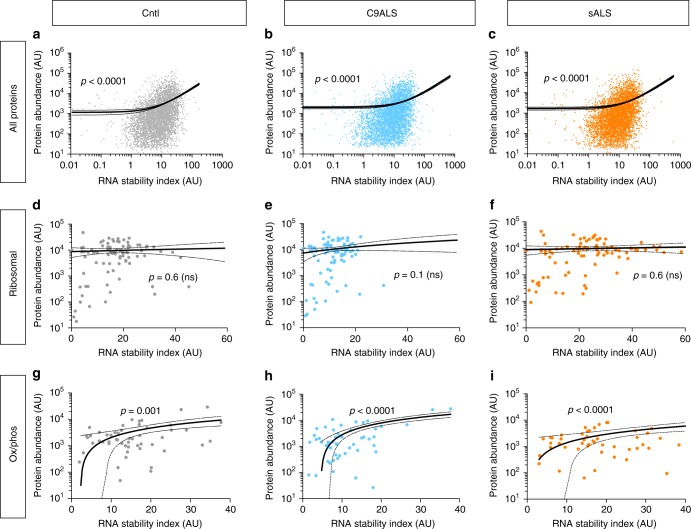
Correlating RNA stability and protein abundance in ALS iPSCs. Linear regression of RNA stability (as measured by the RNA stability index for each transcript, or the abundance at 6 h/0.5 h) and protein abundance determined by MS. A significant (*p* < 0.0001) association was detected between RNA stability and protein abundance in control, C9ALS and sALS iPSCs for all transcripts (top row, **a−c**) and for those involved in oxidative phosphorylation (Ox/phos, bottom row, **g−i**). However, no such relationship was identified for ribosomal protein-encoding transcripts and their corresponding proteins (middle row, **d−f**). Representative scatter plots are shown from one line each of control, C9ALS and sALS iPSCs; identical results were obtained upon examination of other lines. *p* value determined by extra sum-of-squares F test

To examine the relationship between RNA stability and protein levels, we compared RNA stability indices for each transcript to the abundance of the corresponding protein measured by MS. A significant and positive correlation between RNA stability and protein abundance was noted for all datasets (Fig. 7a–c), implying that RNA stability predicts protein concentration, particularly for more stable transcripts. Nevertheless, we were struck by the discrepancy between the relative instability of ribosome protein-encoding RNA in C9ALS iPSCs and the abundance of ribosomal proteins as detected by MS. Specifically, we wondered whether feedback mechanisms may be operating to maintain the concentration of ribosomal proteins^37,38^. In support of this, we did not detect a significant relationship between RNA stability and the abundance of cytoplasmic ribosomal proteins (Fig. 7d–f). In contrast, the stability of oxidative phosphorylation RNAs was clearly tied to the abundance of the corresponding proteins (Fig. 7g–i), consistent with reductions in oxidative phosphorylation proteins in connection with RNA instability in C9ALS fibroblasts and iPSCs.

One hundred and seventy proteins demonstrated concordant changes in RNA stability and protein abundance in C9ALS iPSCs (Supplementary Fig. 15a), representing 18% and 10% of the total changes in RNA stability and protein abundance detected in these cells, respectively. Within this defined set of proteins, we observed a highly significant enrichment for the oxidative phosphorylation pathway (FDR < 1×10^−25^), supporting the proportional relationship between RNA stability and protein concentration for these candidates. In sALS iPSCs, we found 121 proteins displaying concordant changes (Supplementary Fig. 15b), accounting for 14% and 17% of all differences in RNA stability and protein abundance, but no significant enrichment for proteins within a specific functional pathway.

Given the substantial overlap in RNA stability between C9ALS iPSCs and those overexpressing TDP43, we also compared the set of RNAs stabilized or destabilized by TDP-43 overexpression to the set of proteins whose abundance was altered in C9ALS iPSCs. In doing so, we identified 194 proteins with concordant changes in RNA stability and protein concentration (Supplementary Fig. 15c), representing 20% and 11% of the total RNAs and proteins affected in C9ALS iPSCs. Within this set of 194 proteins, components of the oxidative phosphorylation pathway were highly enriched by GO (FDR < 1×10^−21^), consistent with what we observed in C9ALS iPSCs (Fig. 7).

To determine if TDP-43 overexpression could recapitulate the pattern of RNA stability and corresponding changes in protein abundance in C9ALS iPSCs, we separately compared the abundance of this defined set of proteins in C9ALS iPSCs to the stability of their corresponding transcripts in (a) C9ALS iPSCs, or (b) TDP43-overexpressing iPSCs (Supplementary Fig. 15d, e). Linear regression analysis illustrated an analogous relationship between protein abundance in C9ALS iPSCs and RNA stability in C9ALS or TDP43-overexpressing cells, with a slope that was nearly identical in the two groups. This is in contrast to the distinct and steep relationship between RNA stability and protein abundance detected in sALS iPSCs (Supplementary Fig. 15f). These data show that TDP-43 overexpression destabilizes many RNAs whose stability is likewise dysregulated in C9ALS iPSCs, and the proteins corresponding to these RNAs. Furthermore, the transcripts most affected by TDP-43 are highly enriched in components of the mitochondrial oxidative phosphorylation pathway.

### Mitochondria and ribosome function in patient-derived cells

**Fig. 8:**
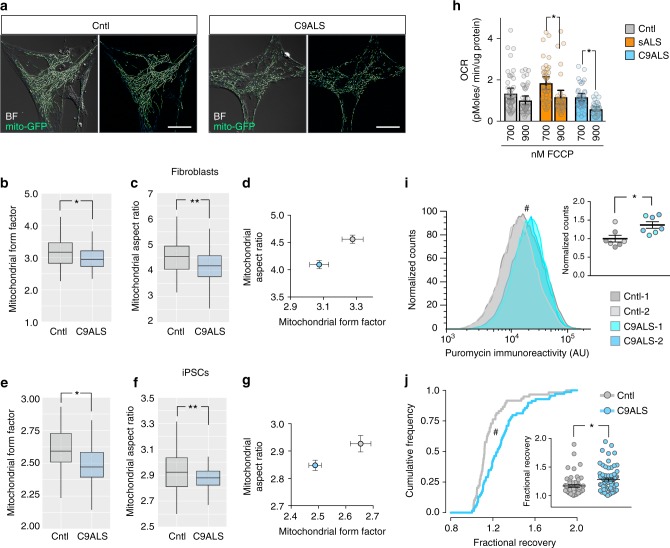
Mitochondria morphology and protein synthesis in ALS patient-derived cells. **a** Mitochondrial morphology in control (Cntl) and C9ALS fibroblasts expressing mito-GFP. BF brightfield. Scale bars = 20 µm. Mitochondrial form factor (a measure of mitochondrial complexity, **b**) and aspect ratio (an estimate of circularity, **c**), or both (**d**) in fibroblasts expressing mito-GFP. Morphological analysis of mitochondrial form factor (**e**), aspect ratio (**f**), or both (**g**) in iPSCs stained with the mitochondrial dye TMRE. *p < 0.05, **p < 0.001 by one-way ANOVA with Tukey’s test. *n* > 20 cells/group. Results in **b−d** were combined from four lines each of control and *C9orf72* fibroblasts, while **e**, **f** were combined from two lines each of control and *C9orf72* iPSCs, performed in duplicate. Plots in **b**, **c**, **e**, **f** show median (horizontal line), interquartile range (box) and maximum/minimum (vertical lines). Graphs in **d** and **g** show mean ± standard error. **h** Bioenergetics analyses demonstrated greater reductions in oxygen consumption rate (OCR) upon addition of 900 nM FCCP, a decoupling agent, to C9ALS and sALS fibroblasts in comparison to controls. *n* = 8 (Control), 8 (sALS), and 7 (C9ALS) lines/group, as described in Supplementary Table 1. Plot in **h** shows mean ± 95% confidence interval. **i** iPSCs from controls and patients carrying *C9orf72* mutations displayed elevated protein synthesis by SUnSET. *p < 0.01 by two-sided Kolmogorov−Smirnov test. Inset shows a scatter plot of normalized anti-puromycin counts (mean ± standard error) from control and C9orf72 mutant iPSCs. *p = 0.0129, unpaired *t* test. *n* = 2 lines each of control and C9ALS iPSCs, in three separate replicates. **j** Cumulative distribution function for fractional recovery of mCherry fluorescence in control and *C9orf72* iPSCs at 3.5 h. *p < 0.01, two-sided Kolmogorov−Smirnov test. Inset illustrates a scatter plot of fractional recovery (mean ± standard error) in control and *C9orf72* mutant iPSCs. *p < 0.01, unpaired *t* test. *n* = 2 lines each of control and C9ALS iPSCs, combined from three replicates

To determine the functional implications of these observations, we assessed mitochondrial and ribosomal activity in ALS patient samples using a variety of methods. First, since mitochondrial function is closely tied to morphology^39,40^, we examined mitochondrial morphology in control and C9ALS fibroblasts by live-cell microscopy (Fig. 8a). Although overall mitochondrial content and length were unchanged (Supplementary Fig. 16a, b), C9ALS fibroblasts displayed relatively simple and rounded mitochondria in comparison to control cells^41^, as determined by mitochondrial form factor (an estimate of irregularity or branching, Fig. 8b) and aspect ratio (representing circularity, Fig. 8c). Accordingly, a composite measure of form factor and aspect ratio clearly distinguished mitochondria from control and C9ALS fibroblast (Fig. 8d). Fixed fibroblasts from sALS and C9ALS patients likewise demonstrated fewer and less distinct mitochondrial puncta in comparison to control cells (Supplementary Fig. 17).

To investigate mitochondrial morphology in iPSCs, we used tetramethylrhodamine (TMRE), a cell-permeable dye that is concentrated within active mitochondria^42^. As in fibroblasts, C9ALS iPSCs demonstrated a reduction in mitochondrial form factor and aspect ratio (Fig. 8e–g) but not mitochondrial content or length (Supplementary Fig. 16c, d), in comparison with control iPSCs. These results closely parallel those obtained in fibroblasts, revealing consistent abnormalities in mitochondrial morphology in C9ALS patient-derived fibroblasts and iPSCs.

We also measured mitochondrial respiratory chain function in control, C9ALS and sALS fibroblasts by Seahorse bioenergetic profiling^43^. Baseline ATP production, coupling efficiency, and ATP-coupled respiration were unchanged in ALS patient fibroblasts (Supplementary Fig. 16e-g). However, we observed differences in spare respiratory capacity when the cells were energetically challenged with higher concentrations of carbonyl cyanide p-[trifluoromethoxy]-phenyl-hydrazone (FCCP) (Fig. 8h). FCCP uncouples the mitochondrial inner membrane by allowing free exchange of ions, thereby depleting the mitochondrial membrane potential^44^. At 900 nM FCCP, both C9ALS and sALS fibroblasts show a significant decrease in spare respiratory capacity, suggesting that these cells are energetically fragile compared to controls and unable to maintain energy production to meet increased energy demands.

To estimate protein synthesis in ALS patient-derived cells, we took advantage of SUnSET (surface sensing of translation)^45^, a nonradioactive and quantitative method that measures the incorporation of puromycin into nascent polypeptides. We were unable to detect a difference in protein synthesis between ALS and control fibroblasts using this method (Supplementary Fig. 16h), which we attributed to the heterogeneity of fibroblasts in culture. However, we detected significantly greater puromycin antibody reactivity in C9ALS iPSCs than in control iPSCs (Fig. 8i), consistent with an increase in overall protein translation in C9ALS iPSCs. To verify these observations, we focused on the kinetics of an exogenous reporter protein (mCherry) expressed under the control of an integrated eIF2 promoter. After a brief photobleach, we measured the fractional return of mCherry fluorescence to estimate the rate of protein synthesis within control and C9ALS iPSCs^46^ (Supplementary Fig. 18). In doing so, we noted a significantly faster rate of return in C9ALS iPSCs compared to control iPSCs (Fig. 8j). These data complement the assessment of protein synthesis by SUnSET and confirm the upregulation of protein synthesis in C9ALS iPSCs that was suggested by proteomics (Fig. 6).

## Discussion

We uncovered consistent abnormalities in RNA stability in fibroblasts and iPSCs from individuals with sporadic and familial ALS due to *C9orf72* mutations. Destabilization of RNAs encoding oxidative phosphorylation and ribosome components was detected in C9ALS fibroblasts and iPSCs, and in control iPSCs overexpressing TDP-43. We also detected reduced abundance of oxidative phosphorylation and ribosomal transcripts in ALS and FTD spinal cord and brain, and in separate RNA-seq datasets from C9ALS iPSCs. This conservation of findings in fibroblasts, iPSCs, and human CNS (Supplementary Table 4) strongly implicates abnormalities in the oxidative phosphorylation and ribosomal pathways in ALS and FTD characterized by TDP-43 pathology.

Age-specific gene expression signatures are lost during the process of reprogramming fibroblasts into iPSCs^47^. Of 60 fibroblast age-related genes mapped in our investigations, 46 (77%) were reversed in iPSCs (Supplementary Table 5), yet we detected prominent changes in RNA stability in C9ALS fibroblasts that were faithfully retained, if not more pronounced, upon reprogramming into iPSCs. Our results therefore suggest that RNA instability in C9ALS likely reflects basic abnormalities in RNA metabolism that are independent of age-related changes in gene expression. Additionally, reprogramming may have eliminated some of the heterogeneity intrinsic to primary fibroblast cultures^26^, thereby enhancing our ability to assess true disease-related phenomena.

We detected pervasive and consistent destabilization of RNAs encoding ribosomal proteins and oxidative phosphorylation components in C9ALS fibroblasts and iPSCs (Supplementary Table 4), but this pattern was less evident in sALS cells, underscoring the heterogeneity of sALS^48^. Even so, and in agreement with previous studies^49^ we observed a reduction in ribosomal and oxidative phosphorylation RNAs in sALS postmortem spinal cord, suggesting that this pattern may eventually emerge over time, or that such abnormalities may be triggered by cell type-specific events unique to neurons and glia of the CNS. The recapitulation of RNA instability in TDP-43 overexpressing iPSCs suggests that TDP-43 accumulation drives RNA destabilization in ALS. In these studies, the effect of TDP-43 overexpression on RNA stability far outweighed that of *C9orf72* mutations, despite the fact that TDP-43 overexpression was limited to 30-50% of transfected iPSCs. In keeping with the strict relationship between TDP-43 levels and cellular survival^15,50^, even relatively minor changes in TDP-43 abundance produced dramatic shifts in RNA stability. Therefore, TDP-43 deposition in the vast majority of individuals with ALS, including C9ALS^5,7^, would be expected to produce similarly significant abnormalities in RNA stability.

Despite the proportional relationship between RNA stability and mitochondrial protein abundance, the stability of ribosomal transcripts was unrelated to their corresponding protein levels. We also detected enhanced rates of protein synthesis in C9ALS iPSCs using two complementary approaches, suggesting functional upregulation of protein translation. Together, these results suggest that C9ALS iPSCs cells compensate for ribosomal RNA destabilization by upregulating translation. This response may be beneficial in the short term, but over time the relative increase in ribosomal subunits may further destabilize ribosomal protein-encoding RNAs through feedback inhibition^37,38^. Moreover, as aberrantly stabilized RNAs are inappropriately translated, cells may rely on proteolytic pathways to maintain homeostasis.

The destabilization of mitochondrial oxidative phosphorylation transcripts and corresponding reduction in protein levels were reflected by subtle abnormalities in mitochondrial morphology and function in ALS fibroblasts and iPSCs. Similar changes in mitochondrial morphology and function are reported in models of ALS^39^, inclusion body myositis^51^, and Parkinson’s disease^52^. TDP-43 binds to and regulates the processing of transcripts encoding mitochondrial proteins^53,54^, and TDP-43 also contains a mitochondrial localizing sequence, leading to mitochondrial dysfunction upon cytoplasmic TDP-43 mislocalization^53^. Our results further emphasize mitochondrial abnormalities in ALS patient-derived cells, and highlight the deleterious effects of TDP-43 deposition on the metabolism of RNAs essential for oxidative phosphorylation.

The two pathways that were primarily affected by RNA instability in ALS patient-derived cells—ribosome biogenesis and oxidative phosphorylation—are crucial for metabolically active cells such as motor neurons that rely heavily on protein translation and mitochondrial function^55,56^. While mitotic cells such as fibroblasts or iPSCs may be able to cope with these abnormalities through cell division, the implications of such abnormalities for post-mitotic and long-lived cells such as neurons may be significantly different.

The relatively stability of transcripts involved in RNA transport in C9ALS iPSCs and TDP43-overexpressing iPSCs may represent a response to nucleocytoplasmic transport abnormalities in ALS^33,57,58^. Indeed, many components of the nuclear pore were stabilized in these cells, as well as *RANGAP1* and *XPO5*, two transport factors implicated in ALS pathogenesis^57,59^. The stabilization of these transcripts may function to counteract deficient RNA export, but based upon the results of previous studies^60,61^, such a response may precipitate neurodegeneration in the long term by facilitating repeat-associated non-AUG (RAN) translation of the *C9orf72* hexanucleotide repeat.

We demonstrated significant alterations in RNA stability in ALS fibroblasts and iPSCs, yet the mechanism responsible for the observed changes remains unknown. Nucleocytoplasmic transport failure in ALS^33,57,58^ may lead to nuclear RNA retention and degradation by the nuclear exosome complex. Alternatively, nuclear splicing factors may be inappropriately mislocalized, resulting in atypical splicing patterns that introduce or remove premature termination codons or affect polyadenylation sites; either of these scenarios could lead to broad changes in RNA stability. A more likely possibility is that the expanded *C9orf72* repeat sequesters essential RBPs that regulate RNA stability. Consistent with this hypothesis, several RBPs preferentially bind expanded nucleotide stretches such as those present in mutant *C9orf72* transcripts^57,62–64^. Many, including ADARB2, hnRNPH and hnRNPA3, are essential for RNA processing, transport, and metabolism^65^. Transcripts demonstrating altered stability in C9ALS and sALS iPSCs are highly enriched in motifs recognized by RBPs that participate in the formation of cytoplasmic stress granules, membrane-less organelles that stabilize non-essential RNAs in the setting of cellular stressors^31,32^. Furthermore, disease-associated *C9orf72* mutations, as well as *TARDBP*, *TIA1*, *FUS*, and *hnRNPA2/B1* mutations, affect stress granule assembly and disassembly^66–70^. Overexpression of TDP-43, an RBP that recognizes many transcripts destined for stress granules^10,12^ and is itself a component of these organelles^71,72^, also affects stress granule dynamics^70,73^. One of the predicted impacts of altered stress granule kinetics is extensive dysregulation of RNA stability, as noted here, but these changes may not be readily apparent without sensitive methods for assessing RNA stability such as BruChase-seq. Lastly, genetic strategies that impact the formation and dissociation of stress granules have pronounced effects on disease outcomes in ALS disease models^66,74^. Together with our observations, these data attest to the promise of therapeutic strategies aimed at restoring RNA homeostasis by preventing RBP sequestration, disruption of RNA granules, and consequent RNA instability. We expect that such strategies, if successful, will not only improve RNA homeostasis, but also prevent the neuron loss and protein deposition that are hallmarks of ALS, FTD and related neurodegenerative disorders.

## Methods

### Study participants

Study participants signed a written informed consent reviewed and approved by the University of Michigan Medical School Institutional Review Board (Protocol # HUM00028826). ALS subjects met the EI Escorial criteria^75^ as determined through the University of Michigan ALS Clinic. Control participants were age-and gender-matched to the ALS participants.

### Fibroblast isolation

Skin punches (3 mm) obtained from ALS and control participants were placed in fibroblast media (FM) (Dulbecco’s modified Eagle medium, DMEM: 4.5 g/l d-glucose, +glutamine, no pyruvate (Gibco/ThermoFisher) supplemented with 10% heat inactivated fetal bovine serum (FBS) (Gibco/ThermoFisher), 1× Glutamax-1 (Gibco/ThermoFisher), and 1× MEM NEAA (Gibco/ThermoFisher)). Tissue was washed 3× with FM, cut into small pieces, resuspended into 500 µl FM and transferred to 2× T-25 flasks, then incubated at 37 °C in 5% CO_2_ for 3 d. Afterwards, the tissue was bathed with 1 ml of fresh FM and incubated for another 3 d. Keratinocytes migrated out of the tissue around d 7 and fibroblast around d 10. The first passage for fibroblasts occurred around d 21 post-isolation. Cells in the parental flasks (T-25 cm^2^) were trypsinized with 0.25% trypsin-EDTA (Gibco/ThermoFisher) and split 1:5 into a 100 mm petri dish (Falcon/Corning) in FM for passage 1. The remaining cells were place in freezing media (FM and 10% DMSO (Sigma-Aldrich)) and separated into five vials. Cells were grown another 7−10 d to reach 85% confluency, trypsinized to make 9 working stock vials in freezing media, and stored in liquid nitrogen in collaboration with the Michigan ALS Consortium at the University of Michigan. Patient-derived fibroblasts used for this project were thawed, grown in FM, and incubated at 37 °C in 5% CO_2_ prior to use. All lines are verified mycoplasma-free on a yearly basis.

### Fibroblast culturing and labeling

Bru-seq and BruChase-seq was performed on 15 lines of fibroblasts—5 lines from controls, 5 from sALS, and 4 from C9ALS patients (Supplementary Table 1). One control line was later excluded due to concern over contamination and poor health. Each fibroblast line was expanded into 2× 150 mm petri dishes (Falcon/Corning) in FM and incubated at 37 °C in 5% CO_2_ until they reached about 80% confluency. Conditioned media was collected from each cell line and used to make 2 mM bromouridine (BrU, Sigma-Aldrich) and 20 mM uridine (Sigma-Aldrich) working solutions. Both plates/line were incubated with 18 ml of 2 mM BrU for 30 min at 37 ^o^C in 5% CO_2_ (pulse). Pulse media was immediately removed and the cells were washed in phosphate-buffered saline (PBS, Gibco/ThermoFisher). For Bru-seq, the cells were harvested with 3 ml of Qiazol (Qiagen) and stored at −80 °C. For BruChase-seq, the washed plate was refed with 18 ml of 20 mM uridine and incubated at 37 °C in 5% CO_2_ (chase) for 6 h. Uridine-containing media was removed, cells were rinsed in PBS, harvested in 3 ml Qiazol and stored at −80 °C prior to RNA purification, as described below.

### Reprogramming and validation of human iPSCs

Fibroblasts were reprogrammed into iPSCs using one of two methods. For two lines (C9A-2 and C9A-4), fibroblasts were transduced with an excisable polycistronic lentiviral vector that carries all four human reprogramming transcription factors (Oct4, Sox2, Klf4, and c-Myc)^27^. The long terminal repeat (LTR) of the virus includes loxP sites that enable the excision of the entire viral genome—including all four reprogramming factors—with Cre recombinase, leaving only a small fragment of the LTR (303 bp) that lacks recognizble cDNA sequences and transcriptional regulatory regions. Successful removal of the reprogramming vector was confirmed by PCR analysis, karyotyping was performed to rule out chromosomal abnormalities, and pluripotency validated by (i) immunocytochemistry for markers of pluripotent stem cells (including Oct-4, TRA-1-60, TRA-1-81, SSEA-3, or SSEA-4), and (ii) differentiation of these lines into three germ layers in vitro and in vivo, confirmed by immunocytochemistry for ß-tubulin III, muscle-actin or desmin and sox-17 for ectoderm, mesoderm and endoderm, respectively (Supplementary Fig. 3).

The second method, utilized for all other iPSC lines, involved transfection of fibroblasts with episomal vectors encoding seven reprogramming factors (Oct4, Sox2, Nanog, Lin28, L-Myc, Klf4, and SV40LT)^28^. We followed the manufacturer’s protocol provided with the Episomal iPSC Reprogramming Vector kit (Invitrogen/ThermoFisher), with the following exceptions: (i) for transfection, 1×10^6^ cells were resuspended in 23 µl instead of 100 µl for transfection; (ii) following transfection, each group of cells was cultured in a single 10 cm plate coated with vitronectin (Gibco/ThermoFisher). No chromosomal abnormalities were noted upon karyotyping, performed with assistance from Cell Line Genetics (Wisconsin, USA; Supplementary Fig. 3). Through the Coriell Institute for Medical Research (Camden, NJ), pluripotency was confirmed using by Pluritest, and further validated by differentiation in vitro into all three germ layers, as determined by quantitative RT-PCR for markers of ectoderm (TP63, KRT14, NOG), mesoderm (RUNX1, PECAM1, TAL1), and endoderm (SOX17, AFP, FOXA2; Supplementary Fig. 3).

### Human iPSC culturing and labeling

All iPSC lines were cultured in E8 media (Gibco/ThermoFisher) on plates coated with vitronectin (diluted 1:100 in PBS, Gibco/ThermoFisher), and passaged every 5–6 d by adding 0.5 mM EDTA (Gibco/ThermoFisher) dissolved in PBS (no Ca/Mg; Gibco/ThermoFisher) followed by gentle trituration in E8 media using a P1000 pipette. All lines are verified mycoplasma-free on a yearly basis. For Bru-seq, we selected two lines each from controls, sALS and C9ALS patients (Supplementary Table 1), and performed all analyses in duplicate. Five 60 mm dishes of 5–6-day-old colonies were used for each condition. Cells were incubated in 1.5 ml of E8 complete media containing 2 mM BrU for 0.5 h at 37 °C. For BruChase-seq experiments, the BrU-containing media was removed, plates were washed one time with PBS (no Mg/EDTA), then incubated for 6 h at 37 °C in 1.5 ml E8 media containing 20 mM uridine. The media was removed, and colonies were washed off the dishes using 750 µl ice-cold Trizol (Sigma-Aldrich). Samples were frozen at −80 °C until RNA extraction, as described below.

For transfection with TDP43-EGFP, cultures were treated with 0.5 mM EDTA for 2 min at 37 °C, colonies dissociated into smaller groups (3–10 cells/colony) by extensive trituration with a P1000 pipette, then plated with Y-27632 (ROCK inhibitor, diluted to 1:100; StemCell Technologies) into a 60 mm vitronectin-coated plate. After overnight incubation at 37 °C, media was replaced with fresh E8. Prior to transfection, 500 µl OptiMEM (Gibco/ThermoFisher) was mixed with 5 µg of total DNA (pGW1-EGFP or pGW1-TDP43-EGFP)^50^ with 7 µl of MirusLT1 transfection reagent (Mirus Bio LLC), and held at room temperature for 20 min. After replacing the E8 media with 2.5 ml mTESR (StemCell Technologies), the DNA/LT1 mix was added to the cells, and the culture kept at 37 °C for 16−20 h, at which time the media was replaced with fresh E8. Three separate transfections of TDP43-EGFP were utilized for Bru-seq and BruChase-seq. Transfection efficiency was assessed at 48 h using an Olympus CKX53 inverted microscope equipped with an SHI-1300L (Olympus) halogen light source. If efficiency was estimated at ≥30%, colonies were harvested for Bru-seq and BruChase-seq as described below.

### Bru-seq and BruChase-seq

Bru-labeled RNA was purified from total RNA using anti-BrU antibodies in a blinded fashion. Strand-specific DNA libraries were then prepared with the Illumina TruSeq Kit (Illumina) and sequenced using the Illumina sequencing platform^24^.

Sequenced data (strand-specific, single-end 52 bp) was first aligned to human ribosomal DNA complete repeating unit (U13369.1) using Bowtie (v0.12.8) and the reads that remained unaligned were mapped to the human genome build hg19/GRCh37 using TopHat (v1.4.1)^24^. Bru-seq (RNA synthesis) data from ALS fibroblasts or iPSCs were compared to control fibroblasts or iPSC samples and fold differences determined using DESeq (version 1.4.1) in R (version 2.15.1). A similar comparison was performed for Bru-seq data from iPSCs transfected with TDP43-EGFP or EGFP alone. Genes having a mean RPKM ≥ 0.5, length ≥ 300 bp, false discovery rate (FDR) ≤ 0.1 and a 1.5-fold change were chosen for downstream bioinformatics analyses.

For BruChase-seq, a stability index for each transcript was calculated as a ratio of transcript abundance at 6 h vs. 0.5 h. To account for stability calculations across replicates for each condition, median values across replicates of the same condition were used. Genes showing greater/less than 1.5-fold change in the stability index were chosen for subsequent analyses. Gene set enrichment analyses based on ontology were accomplished using the Search Tool for the Retrieval of Interacting Genes/Proteins (STRING, https://string-db.org)^25^. All diagrams created using STRING (Figs. 3c, 4d, 6b, d and S9) show confidence, as determined by textmining, experiments, databases, co-expression studies, neighborhood analyses, gene fusions, and co-occurrence. The minimum required interaction score in each case was set to 0.7 (high confidence) or 0.9 (highest confidence). Disconnected nodes were removed from the networks, and structure previews were disabled.

### Mass spectrometry

iPSC cultures were grown for 5–6 d in 60 mm dishes. After application of 0.5 mM EDTA for 2 min, colonies were washed off the plate using 1.5 ml of PBS (no Ca/Mg, Gibco/ThermoFisher), pelleted by centrifugation and stored at −80 °C until lysis. Cell pellets were lysed using 100 mM triethyl ammonium bicarbonate (TEAB; Sigma-Aldrich) with 0.1% SDS by passing the lysate through a 28.5 gauge needle, followed by sonication and centrifugation at 6000 × *g* for 5 min at 4 °C. The supernatant was saved and protein concentration determined by the BCA assay (Pierce). For mass spectroscopy (MS), tandem mass tag (TMT) labeling was performed using the TMT-10plex isobaric labeling kit (Lot#SA239882A; ThermoFisher) according to the manufacturer’s protocol, with minor modifications. A master mix containing equal amount of protein from each sample was created. Sixty-five micrograms of protein from each sample and the master mix were reduced with DTT (5 mM) at 45 °C for 1 h followed by alkylation with 2-chloroacetamide (15 mM) at room temperature for 0.5 h. Proteins were precipitated by adding six volumes of ice-cold acetone and incubating overnight at −20 °C. Precipitated proteins were pelleted by centrifugation at 8000 × *g* for 10 min at 4 °C. The supernatants were discarded, and the pellets resuspended in 100 µl of 100 mM TEAB, then digested overnight at 37 °C by adding 1.1 µg of sequencing grade, modified porcine trypsin (Promega, V5113). TMT reagents were reconstituted in 41 µl of anhydrous acetonitrile and digested peptides were transferred to the TMT reagent vial and incubated at room temperature for 1 h. The TMT channels for each of the samples are provided in Table 1. The reaction was quenched by adding 8 µl of 5% hydroxylamine and incubating for another 15 min. The appropriate samples and master mix (9 samples + 1 master mix) were combined and dried. Prior to MS analysis, two-dimensional separation of the samples was performed. For the first dimension, an aliquot from each sample mix (100 µg) underwent offline fractionation using a high pH reverse phase fractionation kit, following the manufacturer’s protocol (Pierce). Fractions were dried and reconstituted in 10 µl of loading buffer (0.1% formic acid and 2% acetonitrile).

**Table 1:** Mass spectrometry and tandem mass tag (TMT) sample designations

Sample ID	Set	TMT Channel
C1, repl. 1	A	126
C1, repl. 2	A	127N
C1, repl. 3	A	128N
C9A2, repl. 1	A	129N
C9A2, repl. 2	A	130N
C9A2, repl. 3	A	127C
SA2, repl. 1	A	128C
SA2, repl. 2	A	129C
SA2, repl. 3	A	130C
Master Mix	A	131
C4, repl. 1	B	126
C4, repl. 2	B	127N
C4, repl. 3	B	128N
C9A4, repl. 1	B	129N
C9A4, repl. 2	B	130N
C9A4, repl. 3	B	127C
SA4, repl. 1	B	128C
SA4, repl. 2	B	129C
SA4, repl. 3	B	130C
Master Mix	B	131

### Liquid chromatography-mass spectrometry analysis

To increase accuracy and confidence in measures of protein abundance, a multinotch-MS3 method was employed for analyzing MS data^76^. Raw data were acquired using an Orbitrap Fusion (ThermoFisher) and RSLC Ultimate 3000 nano-UPLC (Dionex). Two microliters from each fraction were resolved in the second dimension on a nano-capillary reverse phase column (Acclaim PepMap C18, 2 micron, 75 μm i.d. × 50 cm, ThermoFisher) using a 0.1% formic/acetonitrile gradient at 300 nl/m (2–22% acetonitrile in 150 m; 22–32% acetonitrile in 40 m; 20 min wash at 90% followed by 50 min re-equilibration) and directly sprayed on to Orbitrap Fusion using EasySpray source (ThermoFisher). The mass spectrometer was set to collect one MS1 scan (Orbitrap; 120 K resolution; AGC target 2×10^5^; max IT 100 ms) followed by data-dependent, “Top Speed” (3 s) MS2 scans (collision induced dissociation; ion trap; NCD 35; AGC 5×10^3^; max IT 100 ms). For multinotch-MS3, the top ten precursors from each MS2 scan were fragmented by HCD followed by Orbitrap analysis (NCE 55; 60 K resolution; AGC 5×10^4^; max IT 120 ms, 100−500 m/z scan range).

For MS data analysis, we used Proteome Discoverer (v2.1; ThermoFisher). MS1 and MS2 spectra were queried against the SwissProt human protein database (release 2016-11-30; 42054 sequences) using the following search parameters: MS1 and MS2 tolerance were set to 10 ppm and 0.6 Da, respectively; carbamidomethylation of cysteines (57.02146 Da) and TMT labeling of lysine and N-termini of peptides (229.16293 Da) were considered static modifications; oxidation of methionine (15.9949 Da) and deamidation of asparagine and glutamine (0.98401 Da) were considered variable. A percolator algorithm (PD2.1) was used to determine the FDR and proteins/peptides with FDR ≤ 0.01 were retained for further analysis. Relative quantitation using TMT reporter ions was performed using high-quality MS3 spectra (average signal-to-noise ratio of 10 and <40% isolation interference).

### Bioenergetics (mitochondrial oxygen consumption rate)

Seahorse experiments were performed following the manufacturer’s protocol (Seahorse Bioscience/Agilent). Briefly, XFe96 extracellular flux electrodes were calibrated overnight in 200 µl of sterile water at 37 °C in a CO_2_-free incubator. Approximately 45 min before the bioenergetics assay, water was replaced with 200 µl of XF calibrant buffer pH 7.4 (Seahorse Bioscience /Agilent) pre-warmed at 37 °C. Fibroblasts were plated at a density of 20,000 cells/well in 96-well XFe96 cell culture microplates (Seahorse Bioscience /Agilent) in 100 μl of FM and incubated at 37 °C in 5% CO_2_ for 5 h. One hour before analysis, media was replaced with freshly made serum- and buffer-free assay media (DMEM without glucose, l-glutamine, phenol red, sodium bicarbonate, sodium pyruvate (Corning/Cellgro)) supplemented with 4.5 g/l d-(+) glucose (Sigma-Aldrich), 1× Glutamax-1, 1× MEM NEAA, pH 7.4 and filter-sterilized, and placed in a 37 °C CO_2_-free incubator to equilibrate. Seahorse analysis was performed using final concentrations of 1.2 μM oligomycin (Sigma-Aldrich), 700 nM or 900 nM carbonyl cyanide 4-trifluoromethoxy-phenylhydrazone (FCCP) (Sigma-Aldrich) and 1 μM antimycin A/rotenone (Sigma-Aldrich), which were determined in optimization experiments. The following settings were used: four cycles at rest, 3 cycles/drug injection (mix 3.00, wait 0.00, measure 3.00). After Seahorse analysis, cells were lysed in 25 μM of RIPA buffer (Pierce/ThermoFisher) supplemented with protease inhibitor cocktail tablets (Roche Diagnostics) and total protein/well was determined via BCA (Pierce/ThermoFisher). Data were normalized to total protein and bioenergetics metrics derived from response curves^43^.

### Mitochondrial morphology assessment

Fibroblasts were split at 5000 cells/500 µl of FM, immediately infected with 2 µl of CellLight Mitochondria-GFP, BacMam 2.0 (ThermoFisher), and plated in Lab-Tek II Chambered Coverglass w/cover #1.5 4-well Borosilicate Sterile plates (ThermoFisher). After 72 h of incubation at 37 ^o^C in 5% CO_2,_ the cells were placed in a temperature and CO_2_-controlled chamber (Tokai Hit) for live imaging. Images were acquired on a Nikon A1 confocal microscope with a ×40 oil objective lens (1.3 N.A.) and excited with a 543 nm-wavelength HeNe laser. NIS-Elements Software (Nikon Instruments) was used to acquire images of individual cells at 1024 × 1024 pixel resolution. Approximately 20 images were taken for each cell line and the cell identity was masked from the observer during analysis. Mitochondrial morphology (including form factor and aspect ratio) were determined using a mitochondrial morphology macro^77^ in Fiji.

For analysis of mitochondrial morphology in iPSCs, cultures were grown for 4−5 d after passaging and treated with 200 nM tetramethylrhodamine, ethyl ester (TMRE) directly in E8 media for 15 min at 37 °C. Colonies were washed once with PBS before replacing the media. Imaging was accomplished using a Nikon TiE/B inverted fluorescence microscope with an Andor Zyla 4.2p sCOMS camera, and mitochondrial morphology assessed as above.

### Nonradioactive assessment of protein synthesis with SUnSET

To assess global translation, we used a puromycin-based assay (SUnSET)^45^. Briefly, fibroblast-derived iPSCs were grown in 60 mm dishes with 3 ml of E8 media (Gibco/ThermoFisher) and grown for 4–5 d at 37 °C in 5% CO_2_. The cells were incubated for 20 min with 1 μg/μl puromycin (Sigma-Aldrich) in preconditioned media, lifted with Accutase (StemCell Technologies), and transferred to a 96-well round-bottom plate (Corning). The cells were prepared for intracellular staining following the manufacturer’s protocol (eBioscience), and stained with AlexaFluor^®^ 647 anti-puromycin antibody (Millipore Sigma) or AlexaFluor^®^ 647 mouse IgG isotope control clone MOPC-21 (Biolegend) for 1 h at room temperature. For flow cytometry, cells were resuspended in 100 µl of flow cytometry staining buffer (1× PBS, 2% FBS, 0.1% sodium azide), and transferred to 5 ml polystyrene round-bottom tubes (Corning). Flow cytometry was performed with the assistance of the University of Michigan Flow Cytometry Core using a BD Aria3 cytometer (BD Biosciences). Compensations were determined using single color stained and unstained cells. Median fluorescence intensity was determined for both control and puromycin-stained samples, and expressed as fold change vs. control^45^.

### mCherry photobleaching and fractional recovery

iPSCs were transfected with publicly available vectors for the expression of transcription activator-like effector nucleases (TALENs) targeting the *CLYBL* locus (pZT-C13-R1 and pZT-C13-L1, Addgene) and *a CLYBL* homology cassette (Addgene) containing mCherry under transcriptional control of the eIF2alpha promoter. mCherry-positive colonies were selected using fluorescence microscopy and passaged until all cells displayed homogenous red fluorescence, indicating successful integration of the mCherry cassette. Integration of the mCherry cassette into the *CLYBL* locus was confirmed by qPCR, demonstrating homozygous integration in 2/2 control iPSC lines and heterozygous integration in 2/2 C9ALS lines (data not shown). iPSCs were then grown in an eight-well chamber slide for 3−4 d before bleaching a portion of the colony using a Nikon1-B confocal microscope with a 10x air objective lens and a 543 nm-wavelength HeNe laser, operated via NIS-Elements Software (Nikon Instruments). RFP intensity within the bleached area was normalized to a non-bleached area of equal size at 1 and 3.5 h after bleaching using ImageJ software. Fractional recovery was calculated as the ratio of signal at 3.5 h to that at 1 h, and adjusted for copy number.

### Data availability

Source data for Figs. 1–7 are provided with the paper.

Raw sequencing data have been deposited within the gene expression omnibus (GEO) repository^78^ under the series code GSE115310.

Mass spectrometry proteomics data have been added to the ProteomeXchange Consortium via the PRIDE partner repository^79^ with the dataset identifier PXD009969.

## Electronic supplementary material

Supplementary Information

Description of Additional Supplementary Files

Supplementary Data 1

Supplementary Data 2

Supplementary Data 3

Supplementary Data 4

Supplementary Data 5
