# Supplementary material for: Abnormal RNA stability in amyotrophic lateral sclerosis
Source: Nat Commun. 2018 Jul 20;9:2845. doi: 10.1038/s41467-018-05049-z (PMC6054632; doi:10.1038/s41467-018-05049-z)
Supplement: Supplementary file 1 — Supplementary Information [file 41467_2018_5049_MOESM1_ESM.pdf]

Supplementary information for

Abnormal RNA stability in amyotrophic lateral sclerosis

Author list: Tank EM[1,§], Figueroa-Romero C[1,§], Hinder LM[1], Bedi K[2], Archbold HC[1], Li X[1], Weskamp K[1], Safren N[1], Paez-Colasante X[1], Pacut C[1], Thumma S[1], Paulsen MT[2], Guo K[3], Hur J[3], Ljungman M[2,4], Feldman EL[1,4,5], and Barmada SJ[1,4,5,*]

[§] Authors contributed equally to the work

* To whom correspondence should be addressed: sbarmada@umich.edu

Affiliations:

[1]Department of Neurology, University of Michigan Medical School, Ann Arbor, MI 48109;

[2]Department of Radiation Oncology, University of Michigan Medical School, Ann Arbor, MI 48109;

[3]Department of Biomedical Sciences, School of Medicine and Health Sciences, University of North Dakota, Grand Forks, ND 58202;

[4]Cellular & Molecular Biology Program, University of Michigan Medical School, Ann Arbor, MI 48109;

[5]Neuroscience Graduate Program, University of Michigan Medical School, Ann Arbor, MI 48109.

SUPPLEMENTARY METHODS

RNA extraction and quantitative PCR from brain tissue

90-100 mg total RNA from patient tissue (Supplementary Table 3) was extracted using the RNeasy Lipid Tissue Mini Kit (Qiagen) following the manufacture's protocol. cDNA was synthesized from 1 µg of total RNA using the iScript cDNA Synthesis Kit (Bio-Rad) according to the manufacturer's instructions. The reactions were incubated at 25°C for 5 min, 42°C for 30 min, 85°C for 5 min, and held at 4°C. For quantitative real-time PCR, we used the Step One Plus Realtime PCR system (Applied Biosystems). All reactions were performed using SYBR Green (Applied Biosystems), 200 nM primers, and 0.5 µl cDNA. The thermal cycling conditions included 95°C for 10 min, 40 cycles at 95°C for 15 s, and 60°C for 1 min. Experiments were performed in triplicate for each data point. Relative gene expression was calculated using the comparative Ct (^^Ct) method. Quantitative normalization of cDNA in each sample was performed using GAPDH as an internal reference. GAPDH was one of the housekeeping genes whose synthesis (measured by Bru-seq) and stability (detected by BruChase-seq) did not appreciably change in C9ALS and sALS iPSCs, and was therefore chosen as a reference for quantitative RT-PCR. Although the use of GAPDH as a reference gene is limited by its variability between different tissue types of the same organism[1], here we were applying it as a reference for tissues of the same type, and cells cultured under identical conditions. Primer sequences for PCR and qPCR were as follows:

Target	Forward primer (5’-3’)	Reverse primer (5’-3’)
COX5b	AGG ACA ATA CCA GCG TCG TC	TTG TAA TGG GCT CCA CAG CG
COX6C-X1	CTG CCT CGG ATT TAG TCG TGA	GCG TGA GAG AGT AGA TGC GG

NDUFA13	GGA CCG GAA GTG TGG GAT AC	TCC GTT TGT AGT CGA TGG GC
NDUFA1	GGT AAC GGG GCA GAG ATG TG	GGA TGT ACG CAG TAG CCA GT
RPL28	TCT CGC TCT TGT CGT GTC TG	ATG CCC CGG ATA ATC CTC TG
RPL38	GGA AAC GGA AGT CTC GTT CTT	TCA CCC ACG TAT CAC CCT AGA
RPS18	CTG GAC AAC AAG CTC CGT GA	AGA AGT GAC GCA GCC CTC TA
GAPDH	AAG GTG AAG GTC GGA GTC AA	AAT GAA GGG GTCA TTG ATG G

RNA binding protein motif analysis

Within the 3'UTR transcripts showing a greater/less than 1.5-fold change in the stability, we searched for enrichment of sequence motifs using AME, part of the MEME suite of tools (v 4.1.0)[2]. A total of 24 RNA binding proteins and their corresponding sequence motifs were selected based upon their presence in stress granules, known recognition motifs and genetic linkage to ALS or FTD:

RBP	Motif	Criteria*	References
TDP43	UGUGU	A, B, C	Colombrita C et al. (2009). J Neurochem, 111(4), 1051<br>Polymenidou M et al. (2011) Nat Neurosci, 14, 459<br>Neumann M et al. (2006). Science, 314(5796), 130
FUS	GUGGU	A, B, C	Baron D et al. (2013). Mol Neurodegen, 8(1), 30<br>Lagier-Tourenne C et al. Nat Neurosci, 15(11), 1488<br>Vance C et al. (2009). Science, 323(5918), 1208
TIA1	UUUUU	A, B, C	Dember LM et al. (1996). J Biol Chem, 271;(5)2783<br>Jain M et al. (2016) Cell, 164; 487<br>Mackenzie IR et al. (2017) Neuron, 95(4):808
hnRNPA2/B1	GGUAGUAG	A, B, C	Huelga SC et al. (2012) Cell Rep. 1(2):167<br>Jain M et al. (2016) Cell, 164; 487<br>Kim HJ et al. (2013) Nature, 495(7442), 467
hnRNPA1	UAGGRW	A, B, C	Burd CG and Dreyfuss G (1994) EMBO J. 113;(5)1197<br>Jain M et al. (2016) Cell, 164; 487–498<br>Kim HJ et al. (2013) Nature, 495(7442), 467
FMR1	DWGG	A, B, C	Chen L et al. (2003) Neuroscience 120;(4)1005<br>Jain M et al. (2016) Cell, 164; 487<br>He F et al. (2014) Hum Mol Genet 23(19):5036
RBMS1	DAUAKMS	A, B, C	Ray D et al. (2013) Nature 11;499(7457):172<br>Jain M et al. (2016) Cell, 164; 487<br>Landers et al. (2009) PNAS USA 109(22)9004
SRSF1	RGAAGRRY	A, B, C	Tacke R and Manley JL. (1995) EMBO J. 14;(14)3540<br>Jain M et al. (2016) Cell, 164; 487<br>Hautbergue GM et al. (2017) Nat Comm 8:16063
G3BP2	WAGGAU	A, B	Ray D et al. (2013) Nature 11;499(7457):172<br>Jain M et al. (2016) Cell, 164; 487–498

HuR	UUUDUUU	A, B	Ray D et al. (2013) Nature 11;499(7457):172<br>Jain M et al. (2016) Cell, 164; 487–498
EIF2α	GCAUG	A, B	Ray D et al. (2013) Nature 11;499(7457):172<br>Jain M et al. (2016) Cell, 164; 487–498
hnRNPK	CCAWMCC	A, B	Ray D et al. (2013) Nature 11;499(7457):172<br>Jain M et al. (2016) Cell, 164; 487–498
ELAVL1	UUUGUUU	A, B	Ray D et al. (2013) Nature 11;499(7457):172<br>Jain M et al. (2016) Cell, 164; 487–498
MBNL1	YGCY	A, B	Goers ES et al. (2016) Nucleic Acids Res. 38;(7)2467<br>Jain M et al. (2016) Cell, 164; 487–498
hnRNPAB	RGWYAR	A, B	Ray D et al. (2013) Nature 11;499(7457):172<br>Jain M et al. (2016) Cell, 164; 487–498
FXR1	AYGRMR	A, B	Ray D et al. (2013) Nature 11;499(7457):172<br>Jain M et al. (2016) Cell, 164; 487–498
FXR2	GACRRR	A, B	Ray D et al. (2013) Nature 11;499(7457):172<br>Jain M et al. (2016) Cell, 164; 487–498
PABPC1	AAAAA	A, B	Görlach M et al. (1994) Exp Cell Res. 211;(2)400<br>Jain M et al. (2016) Cell, 164; 487–498
PABPC4	VVAAADV	A, B	Ray D et al. (2013) Nature 11;499(7457):172<br>Jain M et al. (2016) Cell, 164; 487–498
PCBP2	YYYYHCH	A, B	Ray D et al. (2013) Nature 11;499(7457):172<br>Jain M et al. (2016) Cell, 164; 487–498
RBM42	VACUAHD	A, B	Ray D et al. (2013) Nature 11;499(7457):172<br>Jain M et al. (2016) Cell, 164; 487–498
hnRNPH2	GGGAGGG	A, B	Ray D et al. (2013) Nature 11;499(7457):172<br>Jain M et al. (2016) Cell, 164; 487–498
ZNF638	KUKSKD	A, B	Ray D et al. (2013) Nature 11;499(7457):172<br>Jain M et al. (2016) Cell, 164; 487–498
Syncrip	MAAAWW	A, B	Ray D et al. (2013) Nature 11;499(7457):172<br>Jain M et al. (2016) Cell, 164; 487–498

*Criteria: A- component of stress granules; B- known sequence motif; C- associated with ALS or FTD.

Mitochondrial morphology in fixed cells

Patient-derived fibroblast were plated in at a density of 10,000 cells/well in a 96-well plate (Falcon/Corning) in 100 µl of FM and incubated overnight at 37°C in 5% CO_2. Cells were fixed with 2% paraformaldehyde (Sigma-Aldrich) for 10 min at room temperature (RT). Immunohistochemistry was performed using mouse monoclonal anti-mitochondria, surface of intact mitochondria primary antibody, clone 113-1 (Millipore Sigma) diluted to 1:100 in antibody buffer (1X PBS pH 7.4, 0.1% Triton X-100, 1% BSA) overnight at 4°C, followed by secondary incubation with goat anti-mouse Alexa Fluor® 488 antibody at 1:2000 dilution. After removal of secondary antibody, the cells were incubated with monoclonal Cy3 conjugated anti-vimentin

antibody (Sigma-Aldrich) at 1:1000 dilution in antibody buffer for 1 h at RT. The cells were viewed on a Nikon TiE/B inverted fluorescence microscope with an Andor Zyla 4.2p sCOMS camera. Fifty cells/line were highlighted for quantitation by a blinded researcher using ImageJ software. Mitochondrial granularity was estimated on a per-cell basis by determining the coefficient of variation (CV) for each cell, calculated as the ratio of standard deviation in fluorescence intensity to mean fluorescence intensity.

Immunoblotting

iPSCs were rinsed once with PBS, then lysed in RIPA buffer (25mM Tris,150 mM NaCl, 0.1% SDS, 0.5% sodium deoxycholate, 1% Triton X-100) with protease inhibitors (Roche). Lysates were sonicated with a probe sonicator at 30% power for 10 s, and incubated on ice for 30 min prior to centrifugation at 16,000 x g to remove debris. Protein content of the supernatants was determined by the Pierce 660nm Protein Assay. Samples normalized for protein content were run on 4-20% gradient SDS polyacrylamide gels. Proteins were detected using primary antibodies against RPS18 (1:1000, Abcam ab91293), COQ7 (1:500, Proteintech 15083-1-AP), and GAPDH (1:1000, Millipore MAB374), and IR dye-conjugated secondary antibodies (1:10,000, LI-COR Biosciences), imaged on a LI-COR Odyssey, and quantified using ImageStudioLite (LI-COR Biosciences). Plots were created using GraphPad Prism.

Immunocytochemistry

For detection of GR and mitochondria, iPSCs were rinsed twice in PBS and fixed in 4% paraformaldehyde for 10 min. Following 2 more rinses in PBS, the cells were permeabilized with 0.1% Triton X-100 in PBS for 20 min at RT, equilibrated with 10 mM glycine in PBS for 10 min at RT, then blocked in 0.1% Triton X-100, 3% BSA and 0.2% goat serum in PBS for 1 h at RT. Primary antibodies against GR (1:500, rabbit polyclonal, ref. 3) or surface of intact mitochondria (1:100, mouse monoclonal clone 113-1, Millipore Sigma) were added directly to the block before

incubating overnight at 4°C. The cells were rinsed twice quickly and 3 times for 10 min each with PBS, then placed back in block solution containing the appropriate secondary antibodies (goat anti-rabbit Cy5, Jackson ImmunoResearch, (115-175-144), Goat whole IgG (H+L) and Alexa Fluor® 488 goat anti-mouse antibody, Life Technologies) at a dilution of 1:250. The cells were rinsed twice quickly in PBS, and 3 times for 10 min each in PBS containing Hoescht 33342 dye (Invitrogen) at 100 nM, then twice more in PBS before imaging using a Nikon TiE/B inverted fluorescence microscope with an Andor Zyla 4.2p sCOMS camera.

Non-radioactive assessment of protein synthesis using SUnSET

Global translation in fibroblasts was assessed by SUnSET as previously reported[4]. Briefly, patient derived fibroblasts were plated at 2 x 10^5 cells/well in 35 mm tissue culture dishes (Falcon/Corning) and incubated overnight at 37°C in 5% CO_2. The cells were incubated for 20 min with 1μg/μl puromycin (Sigma-Aldrich) in preconditioned media, washed with 1X PBS, trypsinized with 0.25% trypsin-EDTA, and transferred to a 96-well round bottom plate (Corning). Intracellular staining was performed following the manufacture's protocol (eBioscience) and stained with AlexaFluor® 647 anti-puromycin antibody (Millipore Sigma) or AlexaFluor® 647 mouse IgG isotope control clone MOPC-21 (Biolegend) for 1 h at RT. The cells were resuspended in 100 μl of flow cytometry staining buffer (1X PBS, 2% FBS, 0.1% sodium azide) and flow cytometry was performed with the assistance of the University of Michigan Flow Cytometry Core using a BD Aria3 cytometer (BD Biosciences, San Jose, CA, USA). Compensations were determined using single color stained and unstained cells. Median fluorescence intensity was determined for both control and puromycin-stained samples, and expressed as fold change versus control[4].

SUPPLEMENTARY REFERENCES

1. Kozera, B. & Rapacz, M. Reference genes in real-time PCR. *J Appl Genetics* **54,** 391–406 (2013).
2. Bailey, T. L. *et al.* MEME SUITE: tools for motif discovery and searching. *Nucleic Acids Res* **37,** W202–8 (2009).
3. Flores, B. N. *et al.* Distinct C9orf72-Associated Dipeptide Repeat Structures Correlate with Neuronal Toxicity. *PLoS ONE* **11,** e0165084–18 (2016).
4. Schmidt, E. K., Clavarino, G., Ceppi, M. & Pierre, P. SUnSET, a nonradioactive method to monitor protein synthesis. *Nat Meth* **6,** 275–277 (2009).
5. Müller, F.-J. *et al.* A bioinformatic assay for pluripotency in human cells. *Nat Meth* **8,** 315–317 (2011).

Supplementary Table 1

	ID	Bru-Seq	Seahorse	Passage	Age	Sex	Duration (y)	Onset
Control	C1*	✓	✓	4	58	M	–	–
	C2	✓	✓	4	48	M	–	–
	C3	✓	✓	4	48	M	–	–
	C4*	✓	✓	4	52	F	–	–
	C5	✓	✓	4	66	M	–	–
	C6		✓	2	61	M	–	–
	C7		✓	2	80	M	–	–
	C8		✓	2	76	M	–	–
C9ALS	C9A1	✓	✓	4	65	M	1.6	Bulbar
	C9A2*	✓		4	49	M	1.7	Lumbar
	C9A3	✓	✓	4	56	M	1.8	Cervical
	C9A4*	✓		4	52	M	1.9	Lumbar
	C9A5		✓	2	52	M	1.3	Cervical
	C9A6		✓	2	47	M	1.3	Cervical
	C9A7		✓	2	60	M	0.9	Cervical
sALS	SA1	✓	✓	4	52	M	2.5	Lumbar
	SA2*	✓	✓	4	52	M	1.4	Lumbar
	SA3	✓	✓	4	52	M	3.7	Cervical
	SA4*	✓	✓	4	65	F	2.2	Lumbar
	SA5	✓	✓	4	49	M	4.9	Cervical
	SA6		✓	3	80	M	2.6	Lumbar
	SA7		✓	2	77	M	3.2	Lumbar
	SA8		✓	2	48	M	1.7	Lumbar

Supplementary table 1: ALS patient-derived cell lines. Fibroblasts were obtained from the University of Michigan ALS Repository though approved protocols, as described in the Methods section. A subset of these (marked with an *) were reprogrammed into iPSCs for subsequent studies. "Duration" refers to the time in years (y) between onset and biopsy, and "Onset" the site of disease onset

Supplementary Table 2

	Gene	FC	p	Description
C9ALS	ASPM	0.44	0.003	abnormal spindle microtubule assembly
	DACT1	0.45	0.081	dishevelled binding antagonist of beta catenin 1
	ZFPM2	0.49	0.076	zinc finger protein, FOG family member 2
	FOS	3.52	4.9×10^{-6}	Fos AP-1 transcription factor subunit
	WISP2	4.11	1.0×10^{-5}	WNT1 inducible signaling pathway protein 2
	IGFBP5	4.20	1.1×10^{-5}	insulin like growth factor binding protein 5
	CYP7B1	3.14	1.4×10^{-5}	cytochrome P450 family 7 subfamily B-1
sALS	SFRP2	0.18	3.6×10^{-10}	secreted frizzled related protein 2
	FNDC1	0.27	3.6×10^{-6}	fibronectin type III domain containing 1
	DACT1	0.35	5.0×10^{-4}	dishevelled binding antagonist of beta catenin 1
	MKX	0.43	6.3×10^{-4}	mohawk homeobox
	TM4SF1	3.61	5.7×10^{-7}	transmembrane 4 L six family member 1
	MMP1	2.33	0.003	matrix metallopeptidase 1
	GPRC5A	2.46	0.023	G protein-coupled receptor class C group 5-A
	NR4A2	1.97	0.023	nuclear receptor subfamily 4 group A-2

Supplementary table 2: Transcripts demonstrating altered synthesis in C9ALS and sALS fibroblasts. Fold change (FC) and adjusted p value determined using DESeq2

Supplementary Table 3

	ID	Age	Sex	PMI	Tissues
sporadic ALS	1620	67	M	5	Cervical cord, frontal cortex
	1705	77	F	6	Cervical cord, frontal cortex
	1669	66	F	9	Cervical cord, frontal cortex
C9ALS	1666	56	F	11	Cervical cord, temporal cortex
	1729	59	F	17	Cervical cord, temporal cortex
	1693	59	F	12	Cervical cord, temporal cortex
sporadic FTD	1239	62	M	5	Frontal + temporal cortex
	1002	83	M	8	Frontal + temporal cortex
	213	64	F	10	Frontal + temporal cortex
C9FTD	198	56	M	12	Frontal cortex
	470	74	M	10	Frontal cortex
	1335	65	M	11	Frontal cortex
	1233	60	F	5	Frontal cortex
	1077	69	M	6	Frontal cortex
	951	68	F	10	Frontal cortex
Control	196	80	F	19	Cervical cord, frontal + temporal cortex
	729	59	M	12	Frontal + temporal cortex
	90	73	M	7	Frontal + temporal cortex
	1094	100	M	3	Cervical cord
	1432	83	F	21	Cervical cord
	57	74	F	6	Frontal cortex

Supplementary table 3: Post-mortem samples used for qRT-PCR. All samples were obtained from the University of Michigan Brain Bank or ALS Repository. PMI post-mortem interval, in h

Supplementary Table 4

	Fibroblasts	iPSCs	iPSC + TDP43	CNS
**Bru-seq (RNA synthesis)**	C9ALS<br>inflammation	C9ALS<br>ox/phos		
**Bru-seq + BruChase-seq (RNA stability)**	C9ALS<br>ribosome<br>ox/phos	C9ALS<br>ribosome<br>ox/phos	ribosome<br>ox/phos	
**RNA-seq (NeuroLINCS)**		C9ALS<br>ribosome<br>ox/phos		
**qRT-PCR**	C9ALS<br>ribosome<br>ox/phos	C9ALS<br>ribosome<br>ox/phos		C9ALS/FTD<br>sALS<br>ribosome<br>ox/phos
**Proteomics**		C9ALS, sALS<br>ox/phos<br>protein synthesis		
**Function**		C9ALS<br>ox/phos (+sALS)<br>protein synthesis<br>protein degradation		

Supplementary table 4: Conserved changes in ribosome and oxidative phosphorylation pathways in ALS patient-derived samples. Red text color denotes a reduction, while green color signifies an increase in the indicated pathway. Greyed out color represents a non-significant trend. ox/phos oxidative phosphorylation. CNS central nervous system. RNA-seq data were analyzed from the publicly-available NeuroLINCS resource

Supplementary Table 5

Supplementary table 5: Fibroblast aging-related genes displaying concordant changes in RNA stability. Age-related changes in fibroblast gene expression were detected by RNA-seq (*, ref. 47). Age-related changes in RNA stability were determined by comparing the stability of each gene, determined by BruChase-seq, in fibroblasts and iPSCs (#, this study). Concordant changes (46/60 mapped genes, 77%) are indicated in yellow

Aging-related gene*	Gene name	Fold change* (RNA-seq)	Fold change# (BruChase-seq)
KHDRBS3	KH RNA binding domain containing, signal transduction associated 3	0.89	0.55
NETO2	neuropilin and tolloid like 2	3.61	2.4
P2RX5	purinergic receptor P2X 5	1.55	4.44
UCHL1	ubiquitin C-terminal hydrolase L1	1.47	2.7
NES	nestin	2.33	3.78
LYN	LYN proto-oncogene, Src family tyrosine kinase	2.45	2.54
CTSH	cathepsin H	1.64	3.92
SYNGR2	synaptogyrin 2	1.14	2.82
B4GALNT4	beta-1,4-N-acetyl-galactosaminyltransferase 4	1.22	4.16
NEFM	neurofilament, medium polypeptide	2.14	5.39
LRRC8D	leucine rich repeat containing 8 family member D	6.59	1.64
NEFH	neurofilament heavy polypeptide	4.51	3.07
LEPREL1	Leprecan-Like Protein 1	1.35	2.77
SSX2IP	SSX family member 2 interacting protein	5.03	2.94
SEZ6L2	seizure related 6 homolog like 2	1.58	2.8
GNAZ	G protein subunit alpha z	1.6	3.91
SLC2A1	solute carrier family 2 member 1	3.55	2.69
LXN	latexin	1.37	3.56
DOCK8	dedicator of cytokinesis 8	1.09	4.98
ANO4	anoctamin 4	1.95	3.61
C1orf226	chromosome 1 open reading frame 226	4.7	5
RANBP17	RAN binding protein 17	2.38	3.14
PCDH10	protocadherin 10	3.45	4.4
LGALSL	galectin like	2.31	2.78
C3orf70	chromosome 3 open reading frame 70	1.75	3.16
C10orf35	chromosome 10 open reading frame 35	1.23	4.21
ATF7IP2	activating transcription factor 7 interacting protein 2	2.18	3.87
CCDC81	coiled-coil domain containing 81	1.12	3.77
TFAP2A	transcription factor AP-2 alpha	3.23	3.69
PABPC4L	poly A binding protein cytoplasmic 4 like	1.92	4.03
TPI1P2	triosephosphate isomerase 1 pseudogene 2	2.35	1.8
CREB3L1	cAMP responsive element binding protein 3 like 1	7.72	1.31
GPR126	G-Protein Coupled Receptor 126	1.23	2.97
SLC16A4	solute carrier family 16 member 4	3.7	2.97
A2M	alpha-2-macroglobulin	1.01	3.61
SYPL2	synaptophysin like 2	3.06	4.11
LOXL2	lysyl oxidase like 2	1.78	2.4
NTN4	netrin 4	7.09	3.49
GRIK2	glutamate ionotropic receptor kainate type subunit 2	4.11	2.43
LAMA3	laminin subunit alpha 3	1.87	3.32
LOC90246	uncharacterized LOC90246	11.68	4.16
RNF207	ring finger protein 207	2.95	2.98
EREG	epiregulin	5.65	4.54
NPTX1	neuronal pentraxin 1	0.4	0.25
THBS4	thrombospondin 4	0.43	0.12
VWCE	von Willebrand factor C and EGF domains	0.68	0.43
PHEX	Phosphate regulating endopeptidase homolog, X-linked	1.29	0.34
EYA4	Eyes absent homolog 4 (Drosophila)	5.69	0.17
KIF5C	Kinesin family member 5C	0.82	3.3
CELSR2	Cadherin, EGF LAG seven-pass G-type receptor 2 (flamingo	0.56	3.63
FSD1	Fibronectin type III and SPRY domain containing 1	0.95	3.51
ETNK2	Ethanolamine kinase 2	0.62	2.96
APBA2	Amyloid beta (A4) precursor protein-binding, family A, member 2	0.88	4.66
DUSP8	Dual specificity phosphatase 8	0.89	4.07
CORIN	Corin, serine peptidase	0.49	3.32
RGS2	Regulator of G-protein signaling 2, 24kDa	0.19	3.65
CXorf57	Chromosome X open reading frame 57	1.13	0.45
GRM1	Glutamate receptor, metabotropic 1	18.42	0.34
MTRNR2L1	MT-RNR2-like 1	1.1	0.2
ADCY1	Adenylate cyclase 1 (brain)	1.65	0.27

Supplementary Figure 1

a

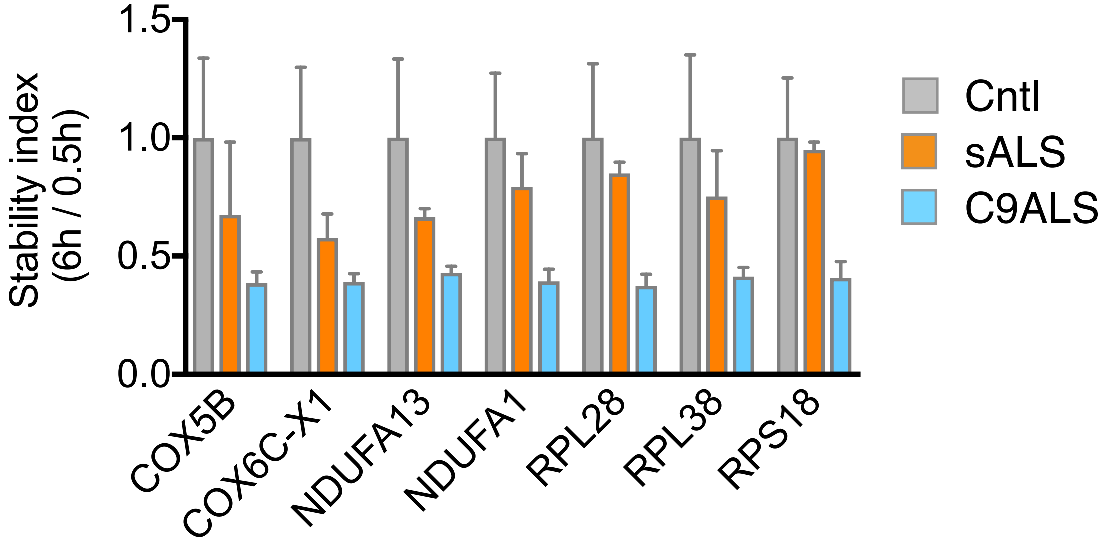

b

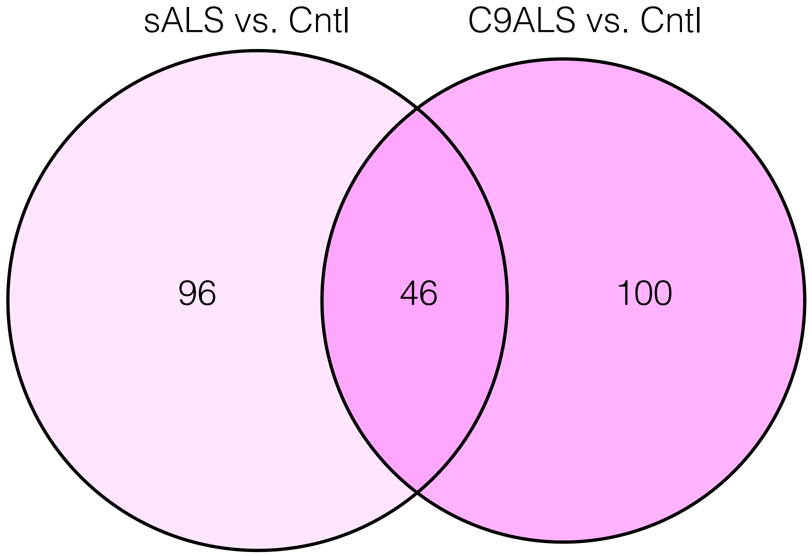

c

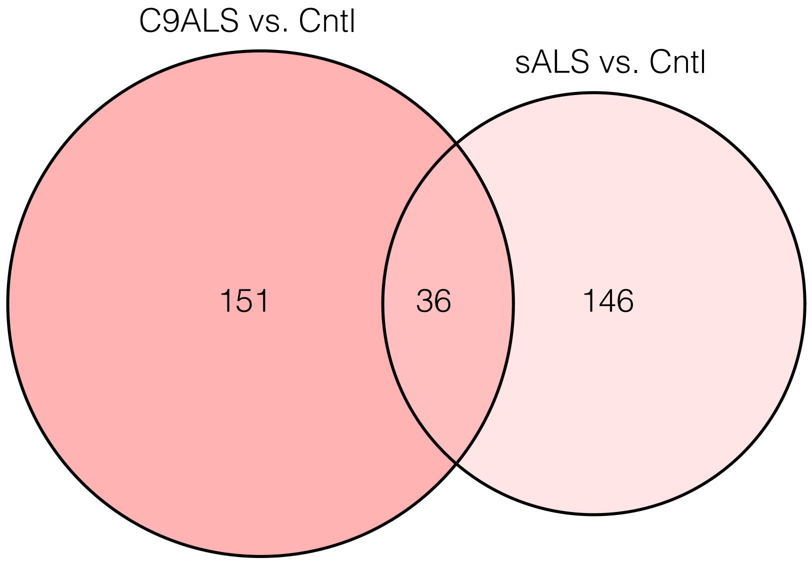

Supplementary figure 1: Altered RNA stability in ALS fibroblasts. a Validation of RNA destabilization in fibroblasts**.** Stability index (ratio of transcript abundance at 6 h / 0.5 h) was compared between control (Cntl), sALS and C9ALS fibroblasts (n = 3 lines (Cntl), 2 lines (C9ALS) and 2 lines (sALS), performed in duplicate). Graph shows mean ± standard error. **b** 46 transcripts showed consistent increases in stability in C9ALS and sALS fibroblasts, compared to control cells, representing 32% (C9ALS) and 35% (sALS) of stabilized transcripts. **c** 36 transcripts showed consistent reductions in stability in C9ALS and sALS fibroblasts, compared to control cells, representing 19% (C9ALS) and 20% (sALS) of destabilized transcripts

Supplementary Figure 2

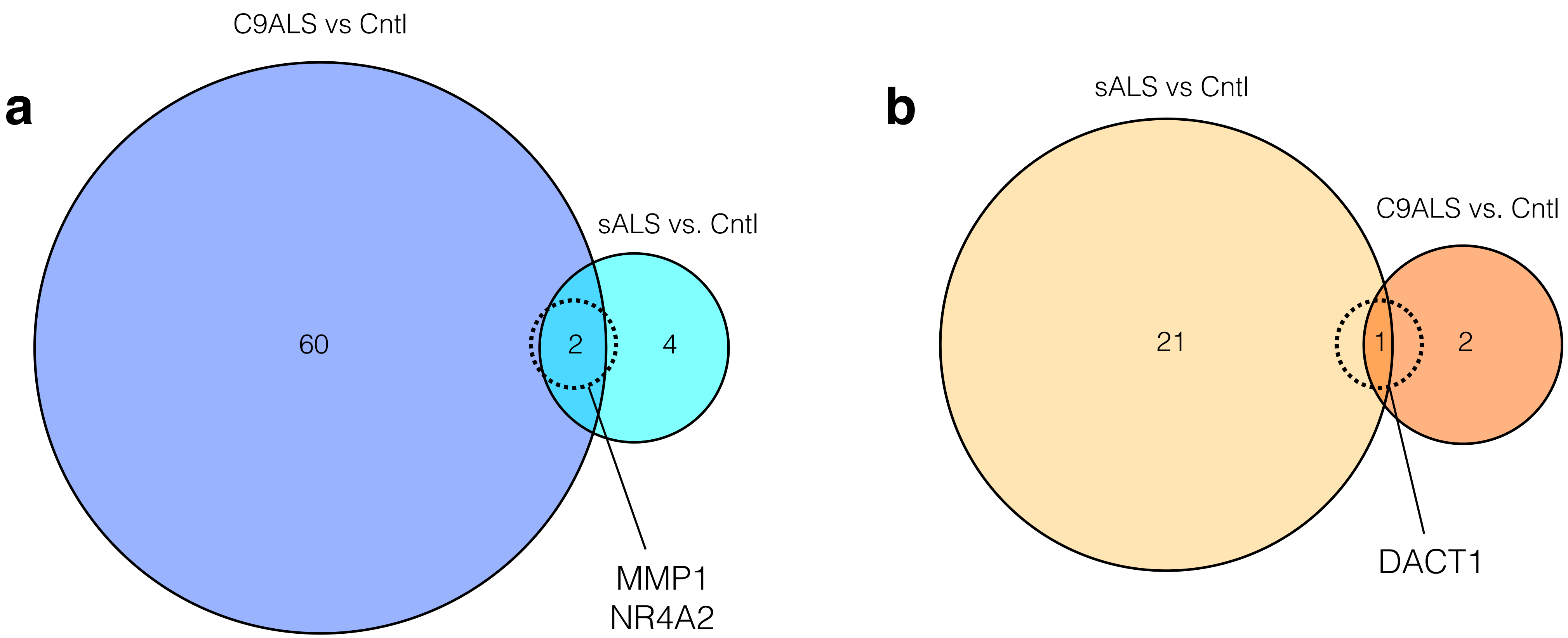

Supplementary figure 2: Venn diagrams for transcripts displaying altered synthesis in C9ALS and sALS fibroblasts. a Two transcripts, MMP1 and NR4A2, demonstrated increased synthesis in C9ALS and sALS fibroblasts, compared to control (Cntl) cells. **b** Only DACT1 displayed reduced synthesis in C9ALS and sALS fibroblasts, compared to control cells

Supplementary Figure 3

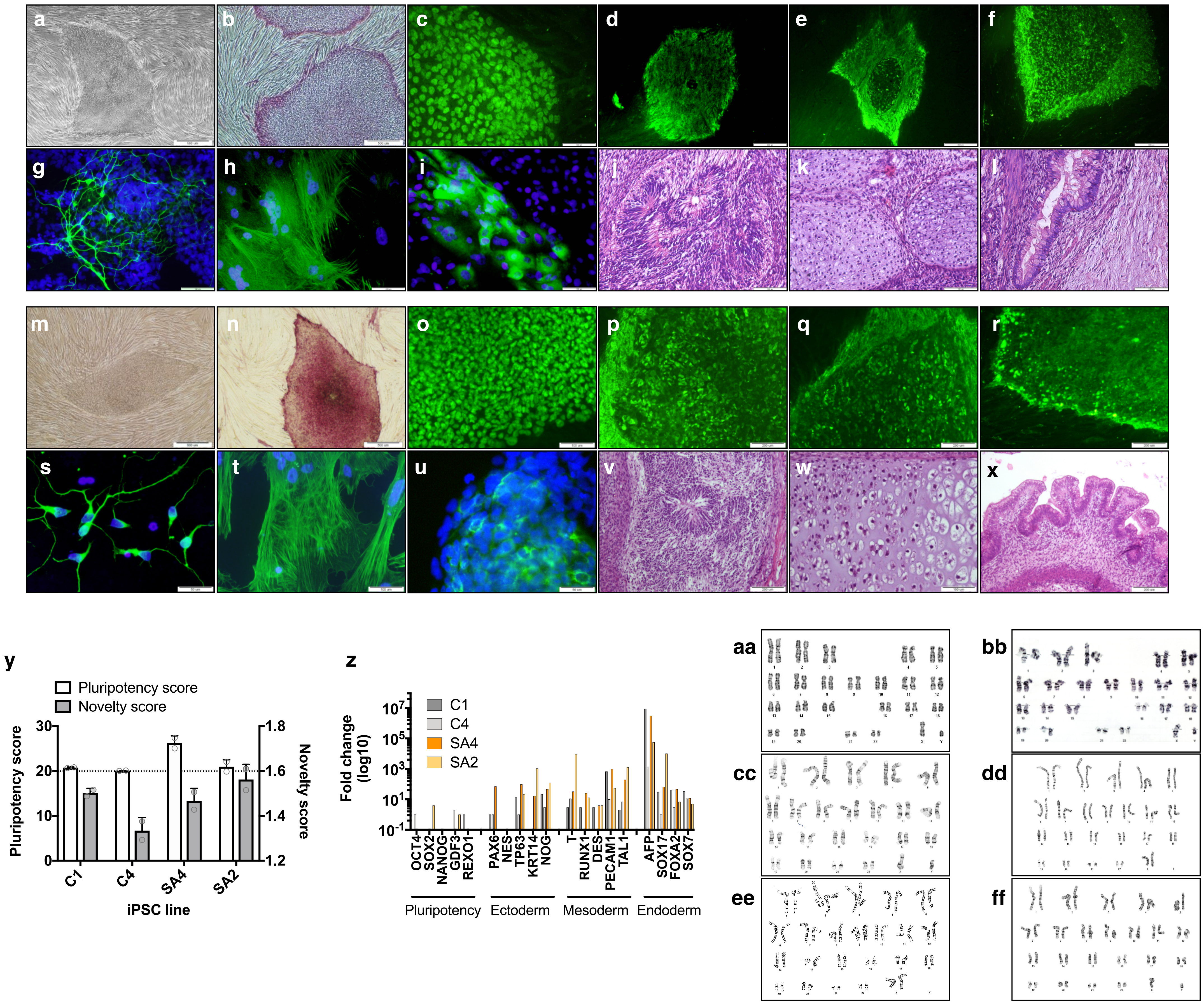

Supplementary figure 3: iPSC characterization. C9A4 (**a-l**) and C9A2 (**m-x**) formed colonies with typical stem cell morphology when cultured on a feeder layer (**a, m**; phase contrast images). Undifferentiated iPS colonies expressed alkaline phosphatase (**b, n**) and Oct-4 (**c, o**). iPS colonies also expressed pluripotent markers Tra-1-60 (**d, p**), Tra-1-81 (**e, q**) and SSEA-4 (**f, r**). Embryoid bodies (EBs) formed from these lines harbored cells expressing neuronal β-tubulin III (**g, s**; ectoderm), muscle actin (**h, t**; mesoderm) and α-fetoprotein (**i, u**; endoderm). Undifferentiated cells were inoculated subcutaneously into NOD SCID mice and teratoma developed. Hematoxylin-eosin stained histological sections of teratoma demonstrated neural rosettes (**j, v**; ectoderm), cartilage/bone (**k, w**; mesoderm) and columnar glandular epithelium (**l, x**; endoderm). Scale bars: 500 µm for a, b, m, n; 200 *µ*m for d, p, e, q, f, r, j, v, l, x; 100 µm for c, o, h, t, k, w; and 50µm for g, s, i, u. **y** Results of the PluriTest[5] in the indicated cell lines, performed in duplicate; Pluripotency scores > 20 and novelty scores < 1.6 indicate less than 10% differentiation. Plot shows mean mean ± standard error. **z** iPSCs were differentiated into EBs, and fold change in gene expression in EBs vs. undifferentiated iPSCs monitored by qRT-PCR. Elevated expression (>2 fold or higher) was noted for genes indicative of all 3 germ layers. Karyotypes were normal for all lines, including C9A4 (**aa**), C9A2 (**bb**), C1 (**cc**), C4 (**dd**), SA4 (**ee**) and SA2 (**ff**)

Supplementary Figure 4

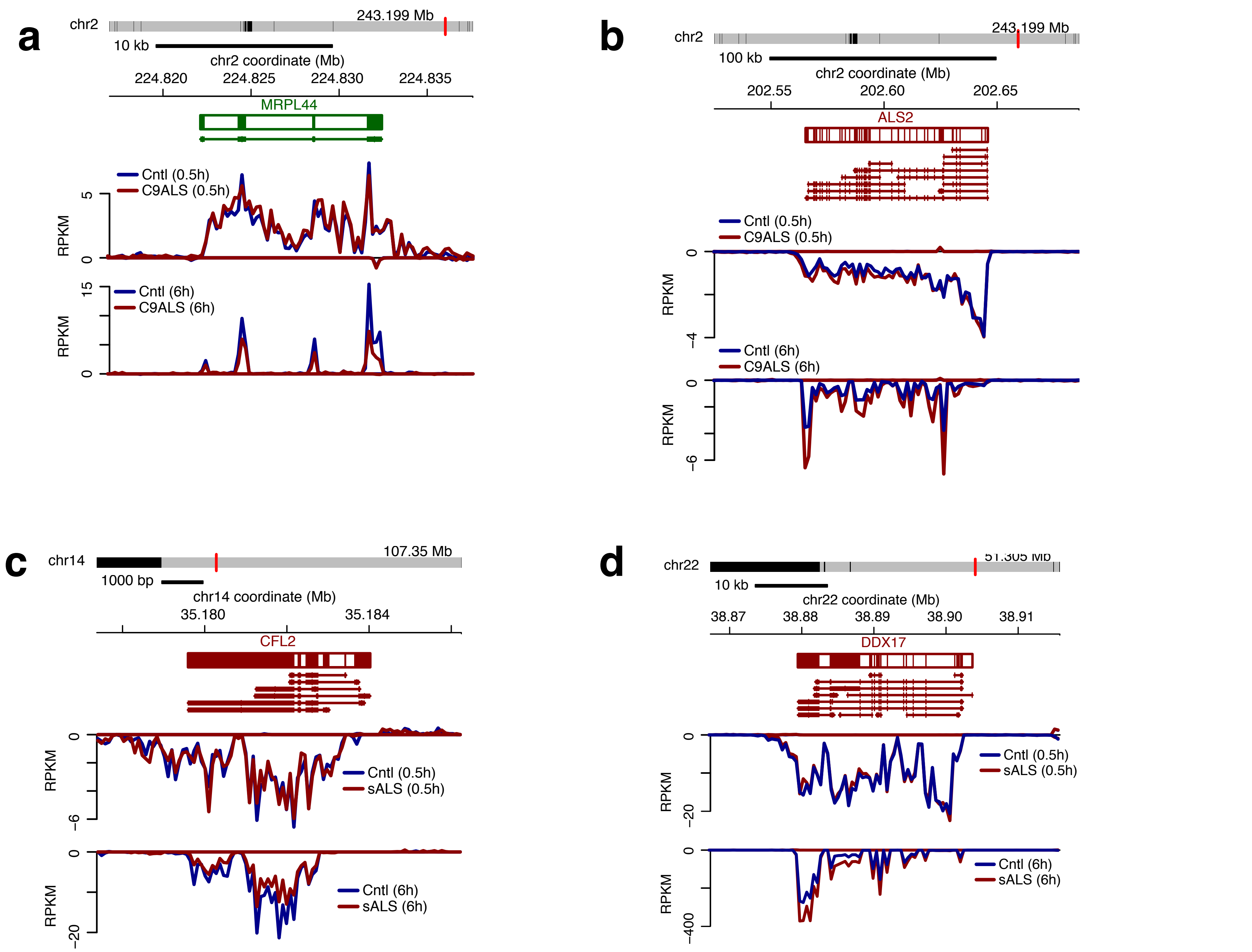

Supplementary figure 4: BruChase-seq in ALS patient-derived iPSCs. Example traces of RNA transcripts showing (**a**) destabilization or (**b**) stabilization in C9ALS iPSCs. Representative examples of RNA transcripts showing (**c**) destabilization or (**d**) stabilization in C9ALS iPSCs

Supplementary Figure 5

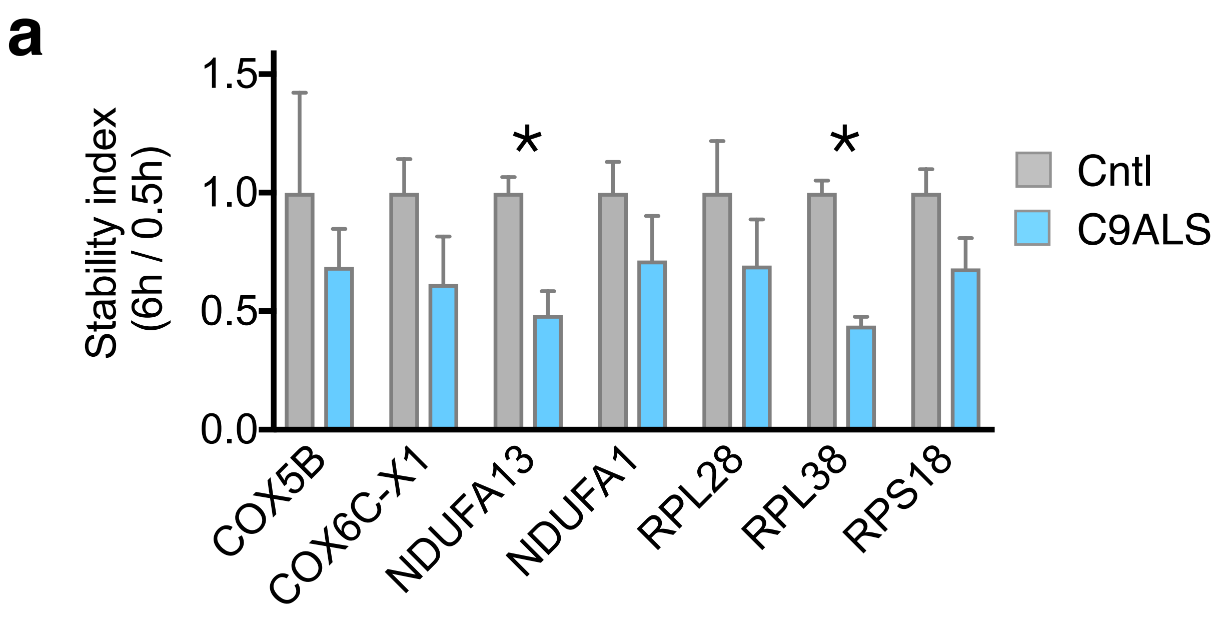

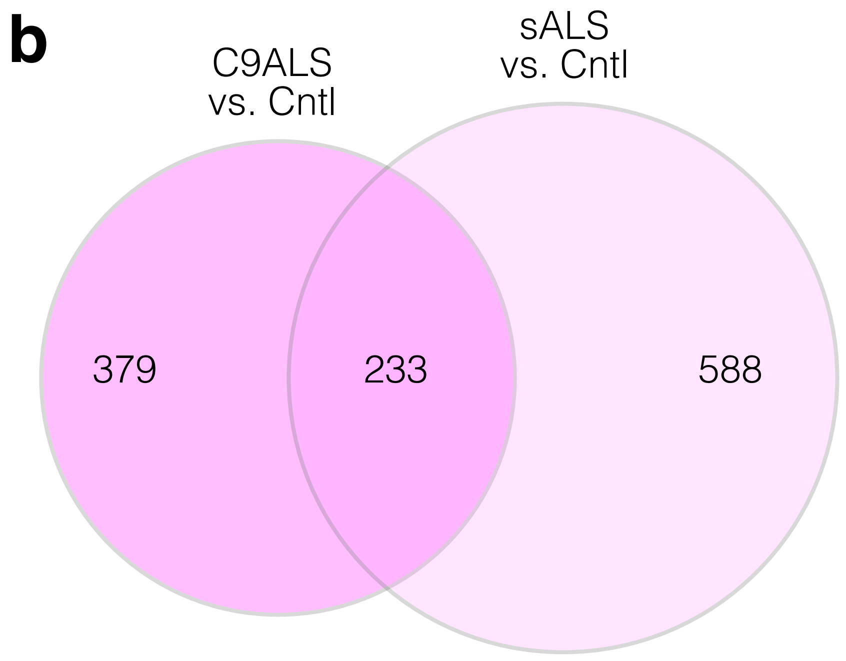

Supplementary Figure 5: Overlap of RNA stability changes in ALS iPSCs. a Validation of RNA stability changes by qRT-PCR. The stability index for each transcript, calculated as the ratio of abundance at 6 h / 0.5 h, highlighted destabilization of select RNAs involved in oxidative phosphorylation and ribosome assembly (n = 2 lines per group, performed in duplicate). *, $p < 0.0001$ by two-tailed t-test. Graph shows mean ± standard error. **b** Venn diagram indicating the 233 transcripts stabilized in both C9ALS and sALS iPSCs, compared to control (Cntl) cells, representing 38% (C9ALS) and 28% (sALS) of stabilized transcripts. **c** 17 transcripts were commonly destabilized in C9ALS and sALS iPSCs, in comparison to control iPSCs, representing 5% (C9ALS) and 39% (sALS) of destabilized transcripts

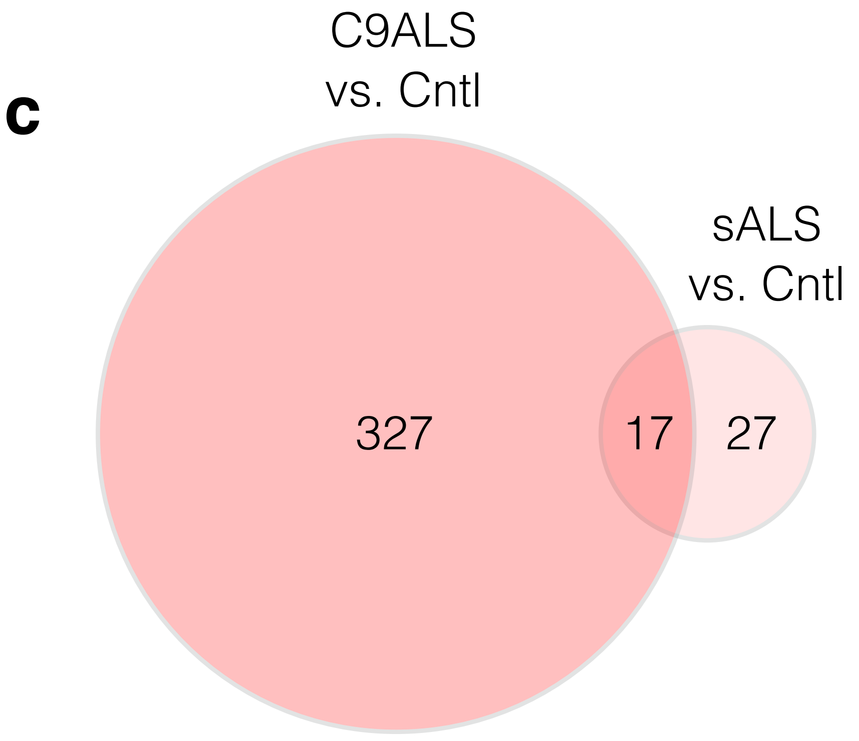

Supplementary Figure 6

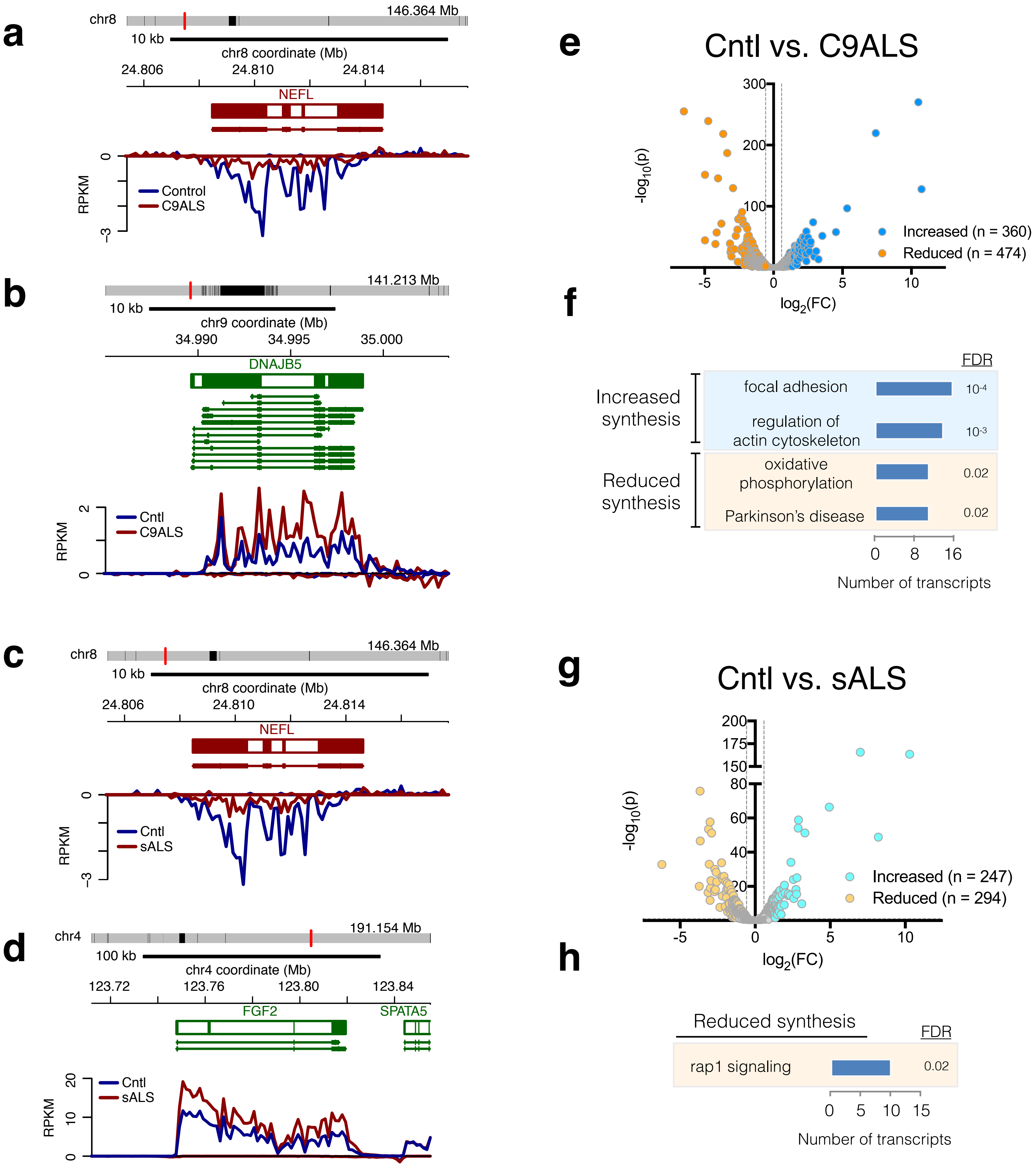

Supplementary figure 6: RNA synthesis in ALS iPSCs. Representative traces of RNA transcripts showing (**a**) reduced or (**b**) increased synthesis in C9ALS iPSCs. Examples of RNA transcripts showing (**c**) reduced or (**d**) increased synthesis in sALS iPSCs. **e** Volcano plot for 834 transcripts showing significant changes in synthesis in C9ALS iPSCs, in comparison to control (Cntl) cells. (**f**) Gene ontology for transcripts displaying altered synthesis in C9ALS iPSCs. FDR, false discovery rate. **g** Volcano plot for 541 transcripts exhibiting significant changes in synthesis in sALS iPSCs, compared to control cells. **h** Gene ontology for transcripts displaying reduced synthesis in sALS iPSCs. FC fold change. Adjusted p value calculated by DESeq2. Dotted lines in (**e**) and (**g**) depict an adjusted p value of 1 and fold change of 1.5

Supplementary Figure 7

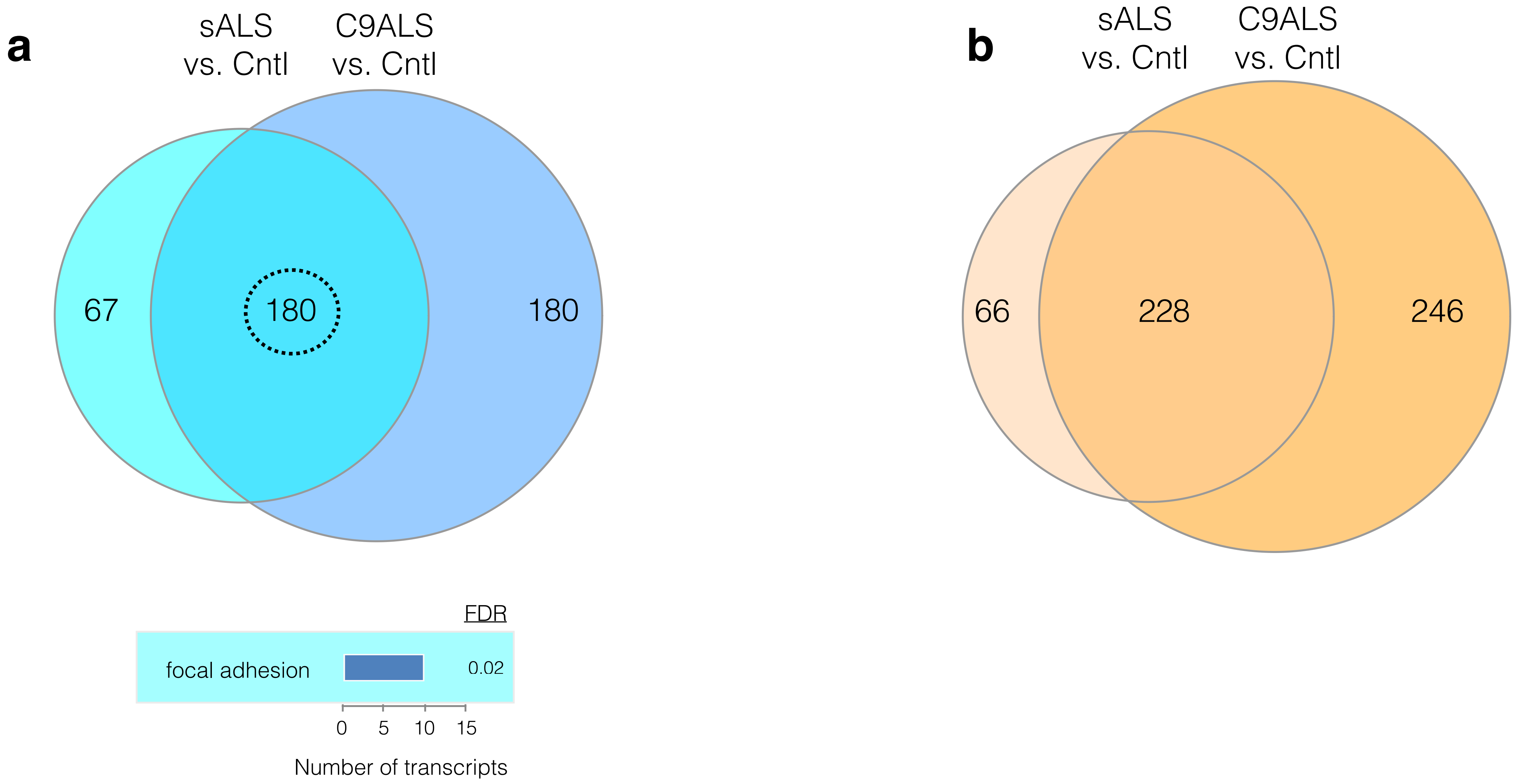

Supplementary figure 7: Pathway analysis of transcripts displaying altered synthesis in ALS iPSCs. a 180 transcripts, enriched in focal adhesion determinants, demonstrated increased synthesis in C9ALS and sALS iPSCs, compared to control (Cntl) cells, representing 50% (C9ALS) and 73% (sALS) of transcripts. **b** 228 transcripts displayed reduced synthesis in C9ALS and sALS iPSCs, compared to control cells, representing 40% (C9ALS) and 80% (sALS) of transcripts, respectively. FDR false discovery rate

Supplementary Figure 8

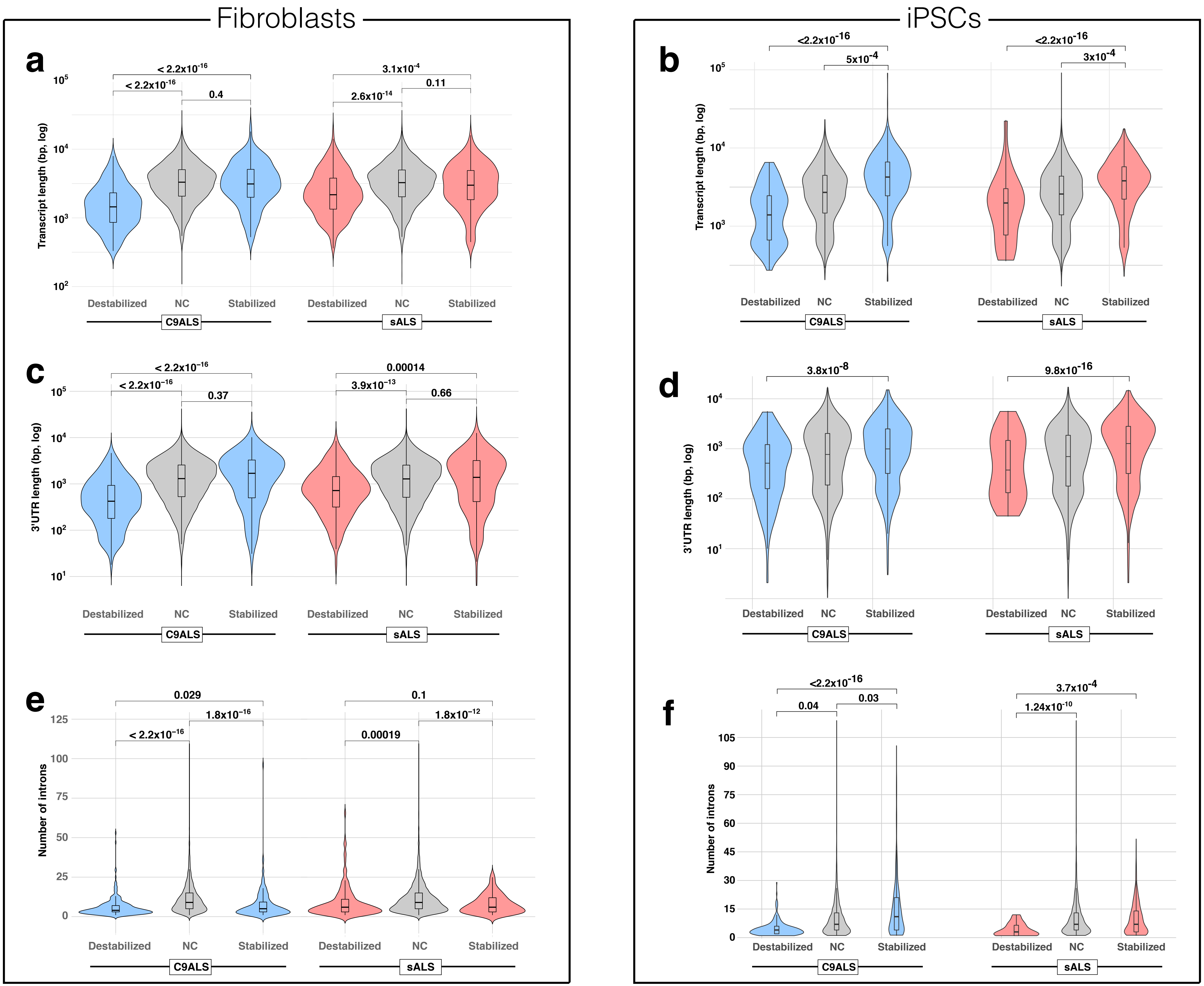

Supplementary figure 8: Features associated with transcript stability. Transcript length is proportional to RNA stability in (**a**) fibroblasts and (**b**) iPSCs from C9ALS and sALS patients, compared to controls. 3'UTR length is also predictive of RNA stability in (**c**) fibroblasts and (**d**) iPSCs from ALS patients. The number of introns per transcript was lower in RNAs that were stabilized and destabilized in ALS (**e**) fibroblasts and (**f**) iPSCs. p values for each comparison determined by Student's t-test for normally distributed data (**a, c, e**) or the Kruskal-Wallis rank-sum test for non-normally distributed data (**b, d, f**). Violin plots show median (horizontal line), interquartile range (box), 95% confidence intervals (vertical lines), and number of observations (width)

Supplementary Figure 9

a Fibroblasts

RBP	Motif	Count	adjusted p-values: fibro_C9_destab	adjusted p-values: fibro_C9_stab	adjusted p-values: fibro_sALS_destab	adjusted p-values: fibro_sALS_stab	-Log10(adjusted p-values): fibro_C9_destab	-Log10(adjusted p-values): fibro_C9_stab	-Log10(adjusted p-values): fibro_sALS_destab	-Log10(adjusted p-values): fibro_sALS_stab	Reference
PCBP2	YYYYHCH	4	7.88E-117	1.61E-77	1.82E-138	9.82E-83	116.1	76.8	137.7	82.0	Ray D et al. Nature. 2013 Jul 11;499(7457):172
PABPC4	VVAAADV	4	5.05E-36	7.47E-52	1.88E-25	3.98E-69	35.3	51.1	24.7	68.4	Ray D et al. Nature. 2013 Jul 11;499(7457):172
huR	UUUDUUU	4	5.57E-33	2.83E-46	7.58E-28	5.69E-56	32.3	45.5	27.1	55.2	Ray D et al. Nature. 2013 Jul 11;499(7457):172
PABPC1	AAAAA	4	2.21E-17	1.44E-35	9.40E-13	1.88E-54	16.7	34.8	12.0	53.7	Görlach M et al. Exp Cell Res. 1994; 211; (2) 400
TIA1	UUUUU	4	2.21E-17	1.44E-35	9.40E-13	1.88E-54	16.7	34.8	12.0	53.7	Ray D et al. Nature. 2013 Jul 11;499(7457):172
ELAVL1	UUUGUUU	4	9.34E-13	6.53E-35	2.41E-11	3.68E-42	12.0	34.2	10.6	41.4	Ray D et al. Nature. 2013 Jul 11;499(7457):172
Syncrip	MAAAWW	4	6.40E-08	9.81E-22	1.94E-04	6.42E-40	7.2	21.0	3.7	39.2	Ray D et al. Nature. 2013 Jul 11;499(7457):172
**TDP43**	**UGUGU**	**4**	**1.91E-06**	**2.64E-22**	**6.51E-22**	**2.38E-30**	**5.7**	**21.6**	**21.2**	**29.6**	Polymenidou M et al. (2011) Nat Neurosci, 14, 459
ZNF638	KUKSKD	4	2.43E-14	2.61E-28	1.48E-26	1.25E-28	13.6	27.6	25.8	27.9	Ray D et al. Nature. 2013 Jul 11;499(7457):172
SRSF1	RGAAGRRY	4	8.59E-27	1.36E-20	6.44E-37	4.33E-16	26.1	19.9	36.2	15.4	Tacke R, Manley JL. EMBO J. 1995; 14; (14) 3540
hnRNPH2	GGGAGGG	4	4.10E-29	9.32E-21	8.60E-60	6.79E-11	28.4	20.0	59.1	10.2	Ray D et al. Nature. 2013 Jul 11;499(7457):172
FMR1	DWGG	4	7.25E-37	9.00E-15	7.25E-66	8.04E-08	36.1	14.0	65.1	7.1	Chen L et al. Neurosci. 2003; 120; (4) 1005
hnRNPK	CCAWMCC	3	5.56E-10	7.09E-07	2.35E-30		9.3	6.1	29.6	0.0	Ray D et al. Nature. 2013 Jul 11;499(7457):172
MBNL1	YGCY	2	6.96E-03		4.36E-15		2.2	0.0	14.4	0.0	Goers ES et al. Nucleic Acids Res. 2010; 38; (7) 2467
FUS	GUGGU	2		2.09E-02	4.89E-11		0.0	1.7	10.3	0.0	Lagier-Tourenne C et al. Nat Neurosci. 2012 15, 1488
EIF2a	GCAUG	3	2.02E-04	2.19E-02	2.63E-10		3.7	1.7	9.6	0.0	Ray D et al. Nature. 2013 Jul 11;499(7457):172
FXR2	GACRRR	1			4.40E-03		0.0	0.0	2.4	0.0	Ray D et al. Nature. 2013 Jul 11;499(7457):172

International Union of Pure and Applied Chemistry (IUPAC) ambiguity code

Code	Represents	Complement
A	Adenine	U
G	Guanine	C
C	Cytosine	G
U	Uracil	A
Y	Pyrimidine (C or U)	R
R	Purine (A or G)	Y
W	weak (A or U)	W
S	strong (G or C)	S
K	keto (U or G)	M
M	amino (C or A)	K
D	A, G, U (not C)	H
V	A, C, G (not U)	B
H	A, C, U (not G)	D
B	C, G, U (not A)	V
X/N	any base	X/N

b iPSCs

RBP	Motif	Count	adjusted p-values: iPSC_C9_destab	adjusted p-values: iPSC_C9_stab	adjusted p-values: iPSC_sALS_destab	adjusted p-values: iPSC_sALS_stab	-Log10(adjusted p-values): iPSC_C9_destab	-Log10(adjusted p-values): iPSC_C9_stab	-Log10(adjusted p-values): iPSC_sALS_destab	-Log10(adjusted p-values): iPSC_sALS_stab	Reference
huR	UUUDUUU	3	3.64E-10	2.92E-31		1.94E-38	9.4	30.5	0.0	37.7	Ray D et al. Nature. 2013 Jul 11;499(7457):172
PCBP2	YYYYHCH	3	1.01E-04	7.18E-15		2.09E-22	4.0	14.1	0.0	21.7	Ray D et al. Nature. 2013 Jul 11;499(7457):172
PABPC4	VVAAADV	3	2.01E-02	4.12E-11		1.46E-21	1.7	10.4	0.0	20.8	Ray D et al. Nature. 2013 Jul 11;499(7457):172
TIA1	UUUUU	3	2.78E-04	6.91E-13		3.80E-18	3.6	12.2	0.0	17.4	Ray D et al. Nature. 2013 Jul 11;499(7457):172
PABPC1	AAAAA	3	2.78E-04	6.91E-13		3.80E-18	3.6	12.2	0.0	17.4	Görlach M et al. Exp Cell Res. 1994; 211; (2) 400
ELAVL1	UUUGUUU	3	1.08E-02	2.01E-12		1.18E-15	2.0	11.7	0.0	14.9	Ray D et al. Nature. 2013 Jul 11;499(7457):172
**TDP43**	**UGUGU**	**3**	**2.00E-02**	**1.65E-12**		**3.63E-13**	**1.7**	**11.8**	**0.0**	**12.4**	Polymenidou M et al. (2011) Nat Neurosci, 14, 459
Syncrip	MAAAWW	2		7.88E-08		7.54E-10	0.0	7.1	0.0	9.1	Ray D et al. Nature. 2013 Jul 11;499(7457):172
hnRNPH2	GGGAGGG	2		3.33E-06		2.86E-07	0.0	5.5	0.0	6.5	Ray D et al. Nature. 2013 Jul 11;499(7457):172
FXR1	AYGRMR	2		1.89E-04		4.55E-05	0.0	3.7	0.0	4.3	Ray D et al. Nature. 2013 Jul 11;499(7457):172
SRSF1	RGAAGRRY	1				2.09E-04	0.0	0.0	0.0	3.7	Tacke R, Manley JL. EMBO J. 1995; 14; (14) 3540
FXR2	GACRRR	1				3.72E-03	0.0	0.0	0.0	2.4	Ray D et al. Nature. 2013 Jul 11;499(7457):172

Supplementary figure 9: Stress granule RBP motifs are enriched in the 3'UTR of transcripts whose stability is affected in ALS patient derived cells. AME was used to determine if the 3'UTR of transcripts demonstrating stability changes in ALS fibroblasts (**a**) or iPSCs (**b**) were enriched for motifs recognized by any of 24 RNA binding proteins (RBPs) that (*i*) possess an experimentally-derived sequence recognition motif; (*ii*) exhibit functional or genetic linkage to ALS or FTD; and (*iii*) are involved in stress granule assembly. A full list of these RBPs and their recognition motifs is provided in the Supplementary Methods section. Darker shading corresponds to higher statistical significance, as determined by the Wilcoxon rank-sum test with Bonferroni correction (adjusted p value)

Supplementary Figure 10

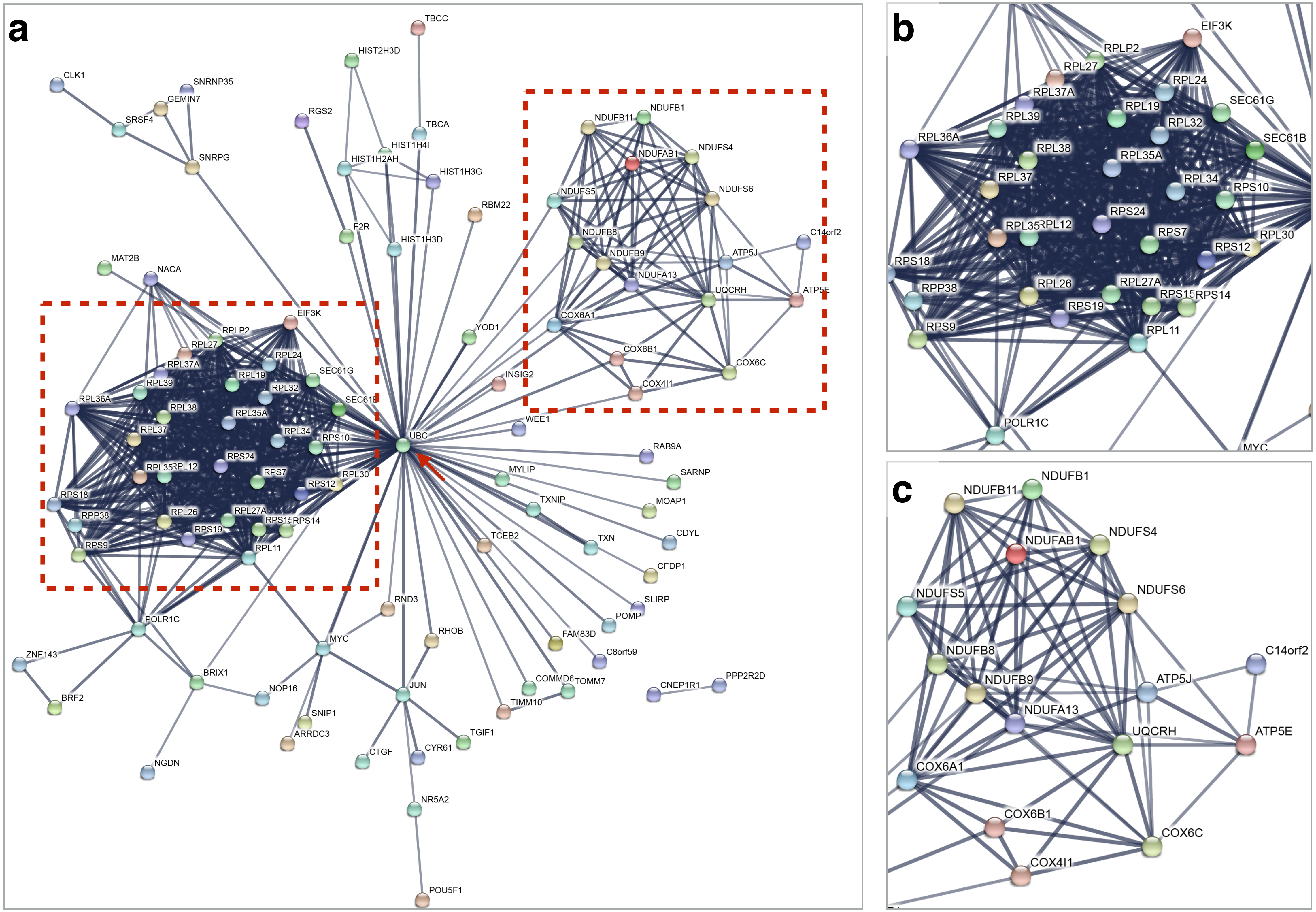

Supplementary figure 10: STRING network analysis of transcripts displaying altered stability in ALS and TDP43-EGFP overexpressing iPSCs. Higher magnification views of (**a**) are shown in (**b**) and (**c**). Arrow in (**a**) denotes ubiquitin (UBC)

Supplementary Figure 11

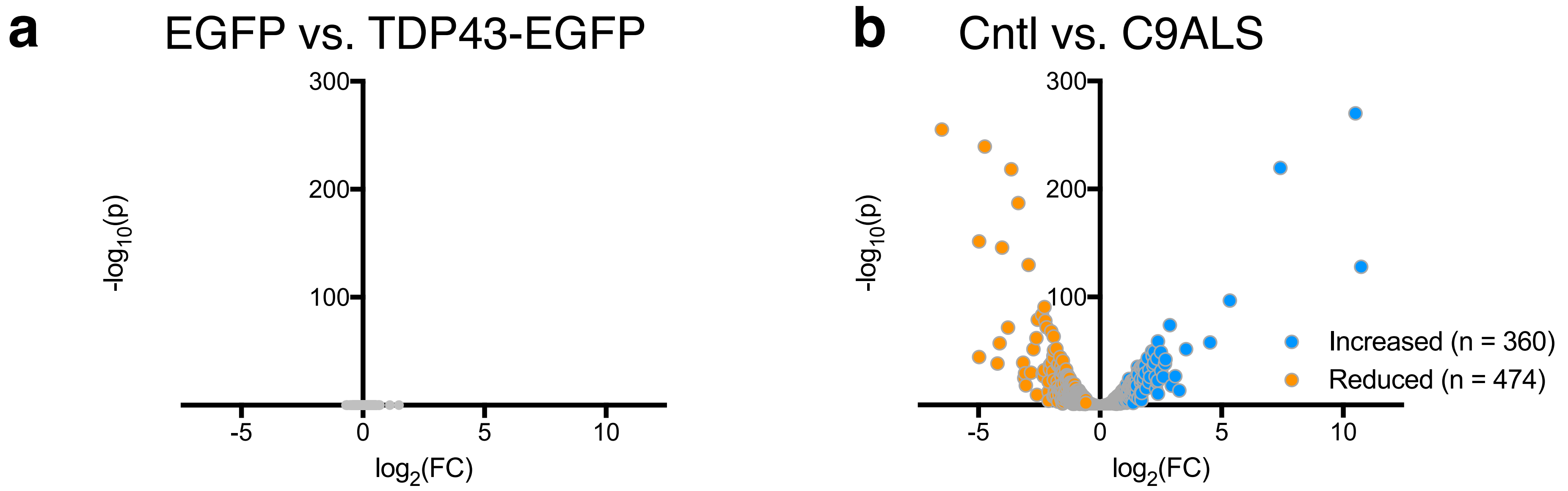

Supplementary figure 11: TDP43-EGFP overexpression has no detectable effect on RNA synthesis. a No RNAs demonstrated a significant change in synthesis rates in TDP43-EGFP overexpressing iPSCs, in comparison to cells transfected with EGFP, by Bru-seq. **b** As a comparison, volcano plot showing changes in RNA synthesis in C9ALS iPSCs vs. control iPSCs, from Supplementary Fig. 6e

Supplementary Figure 12

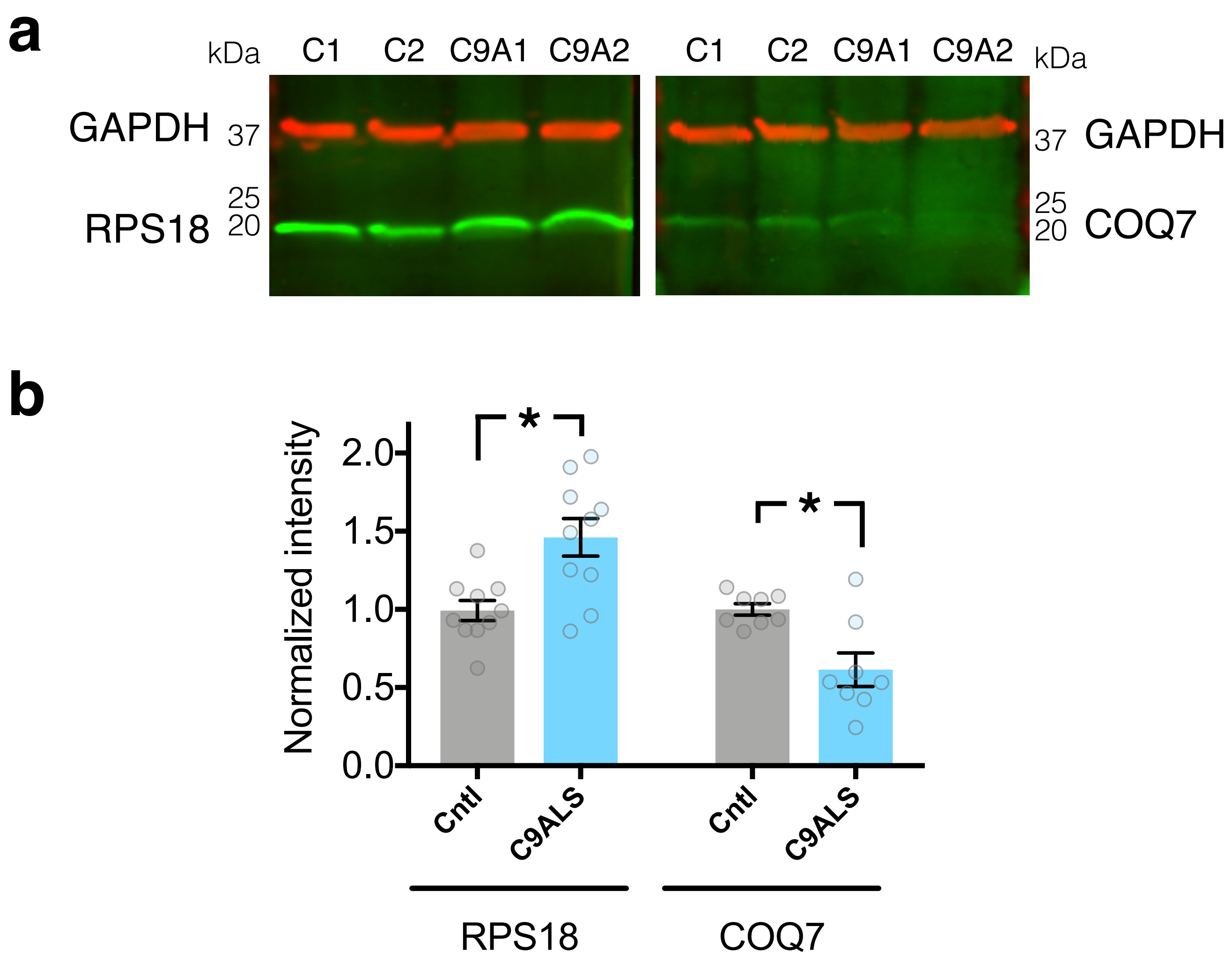

Supplementary figure 12: Confirmation of changes for select proteins in C9ALS iPSCs. a Representative immunoblots from control (C1, C2) and C9ALS (C9A1, C9A2) iPSCs detected using antibodies recognizing RPS18 and COQ7, components of cytoplasmic ribosomes and the mitochondrial oxidative phosphorylation machinery, respectively. **b** Quantitative abundance of RPS18 and COQ7 in control (Cntl) and C9ALS iPSCs, normalized to values obtained from control iPSCs. Plot shows mean ± standard error, determined from 3 biological replicates of 2 iPSC lines per group.

Supplementary Figure 13

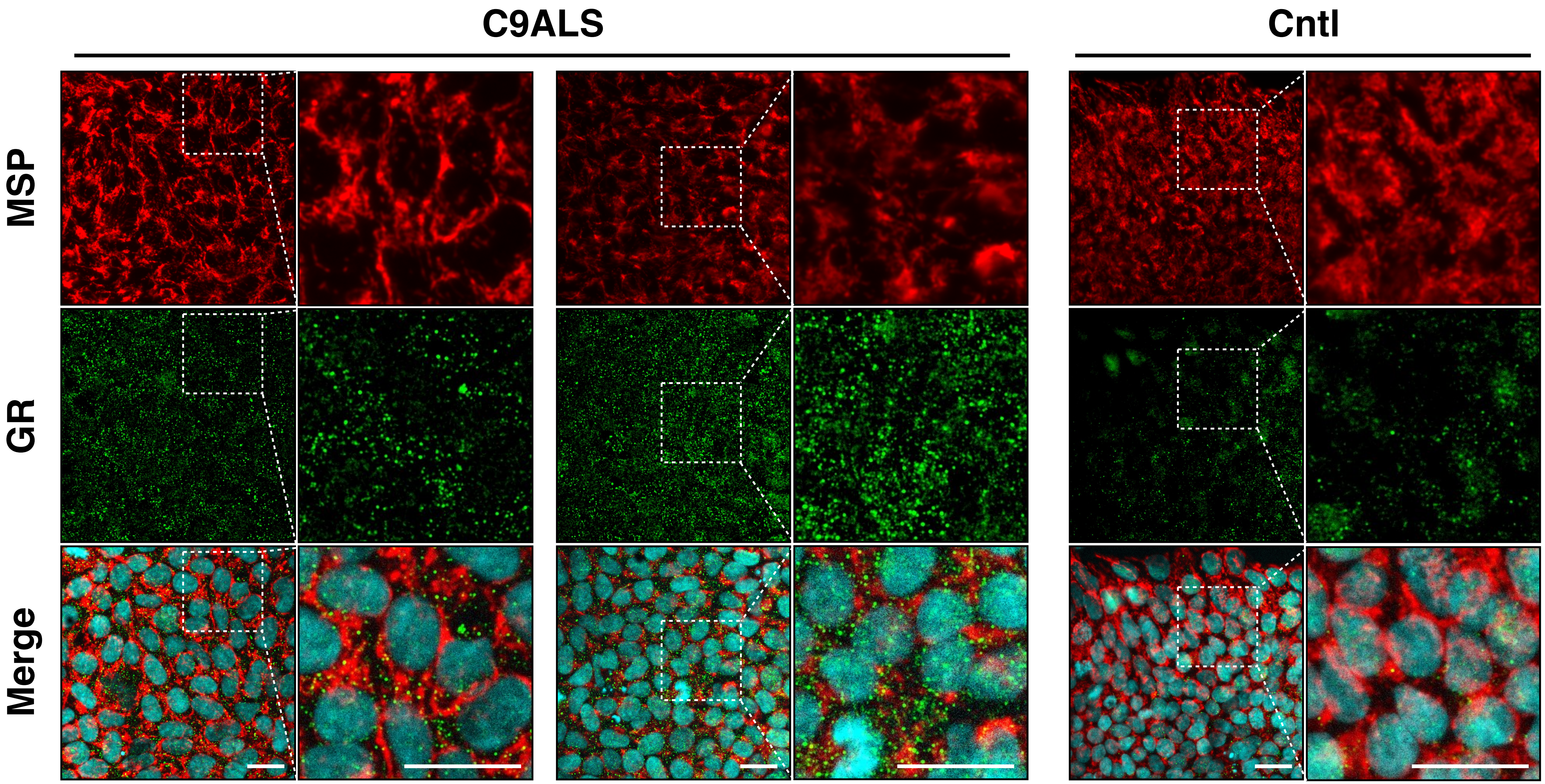

Supplementary figure 13: Glycine-arginine dipeptide repeat proteins do not colocalize with mitochondria in C9ALS iPSCs. C9ALS and control (Cntl) iPSCs were stained with antibodies against mitochondrial surface protein (MSP, red) and glycine-arginine (GR, green) dipeptide repeat proteins. All cells were co-stained with DAPI to mark the nuclei (blue). Dotted boxes indicate regions viewed at higher magnification. Scale bar, 50 μm

Supplementary Figure 14

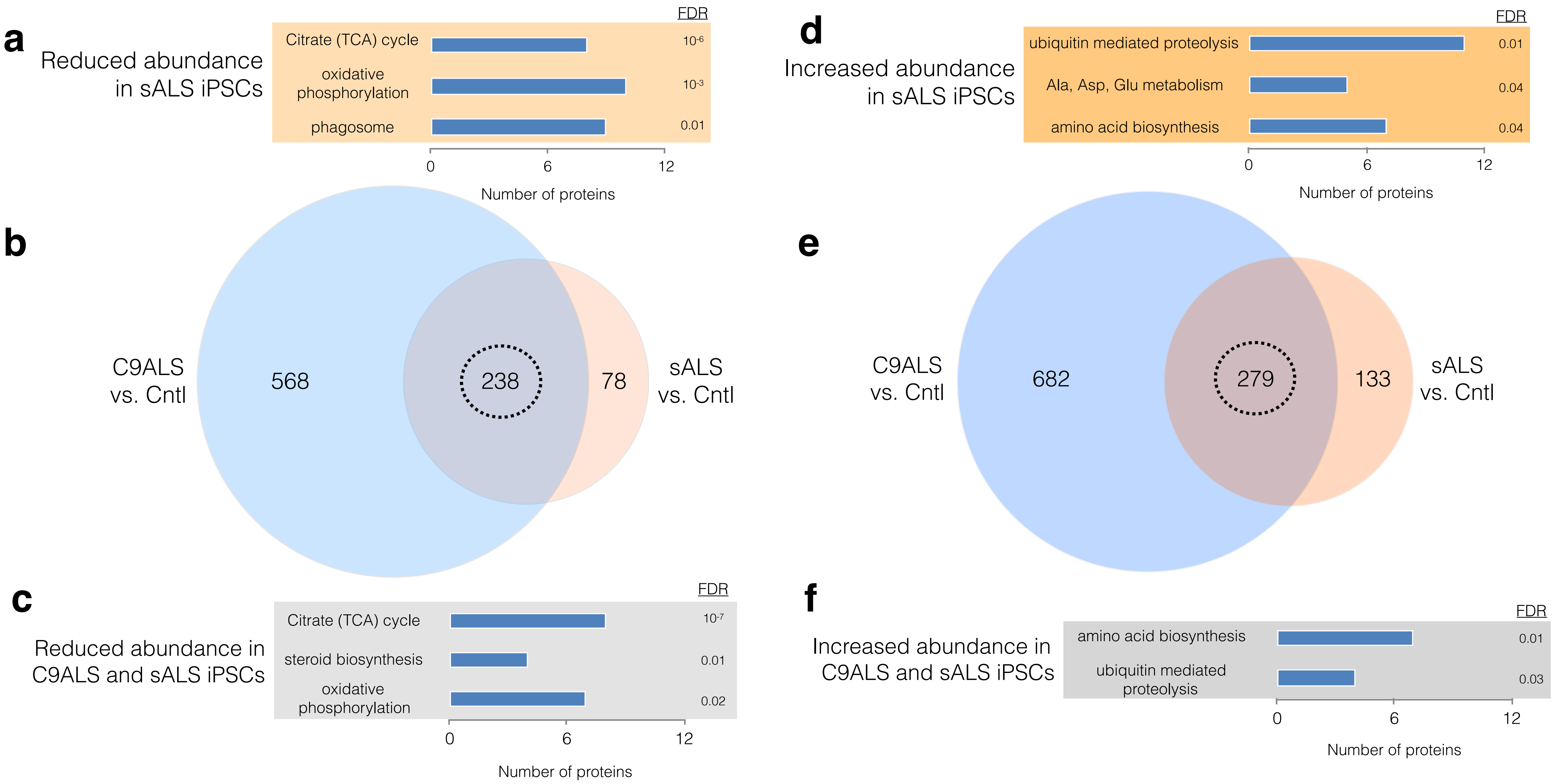

Supplementary figure 14: Conserved patterns in sALS and C9ALS iPSC proteomics. a Gene ontology for 316 proteins reduced ≥ 10% in sALS iPSCs, compared to controls. **b** Venn diagram illustrating the overlap among proteins reduced in both C9ALS and sALS iPSCs. The 238 proteins commonly reduced in abundance represent 30% (C9ALS) and 75% (sALS) of reduced proteins. (**c**) Gene ontology for the overlapping 238 proteins in (**b**), highlighting enrichment in metabolic and oxidative phosphorylation pathways. **d** Gene ontology for 412 proteins increased ≥ 10% in sALS iPSCs, compared to control (Cntl). **e** Venn diagram showing 279 proteins commonly increased in C9ALS and sALS iPSCs, compared to controls, representing 29% (C9ALS) and 68% (sALS) of increased proteins. **f** Gene ontology for 279 proteins circled in (**e**), emphasizing enrichment in amino acid biosynthesis and ubiquitin-mediated proteolysis pathways.

Supplementary Figure 15

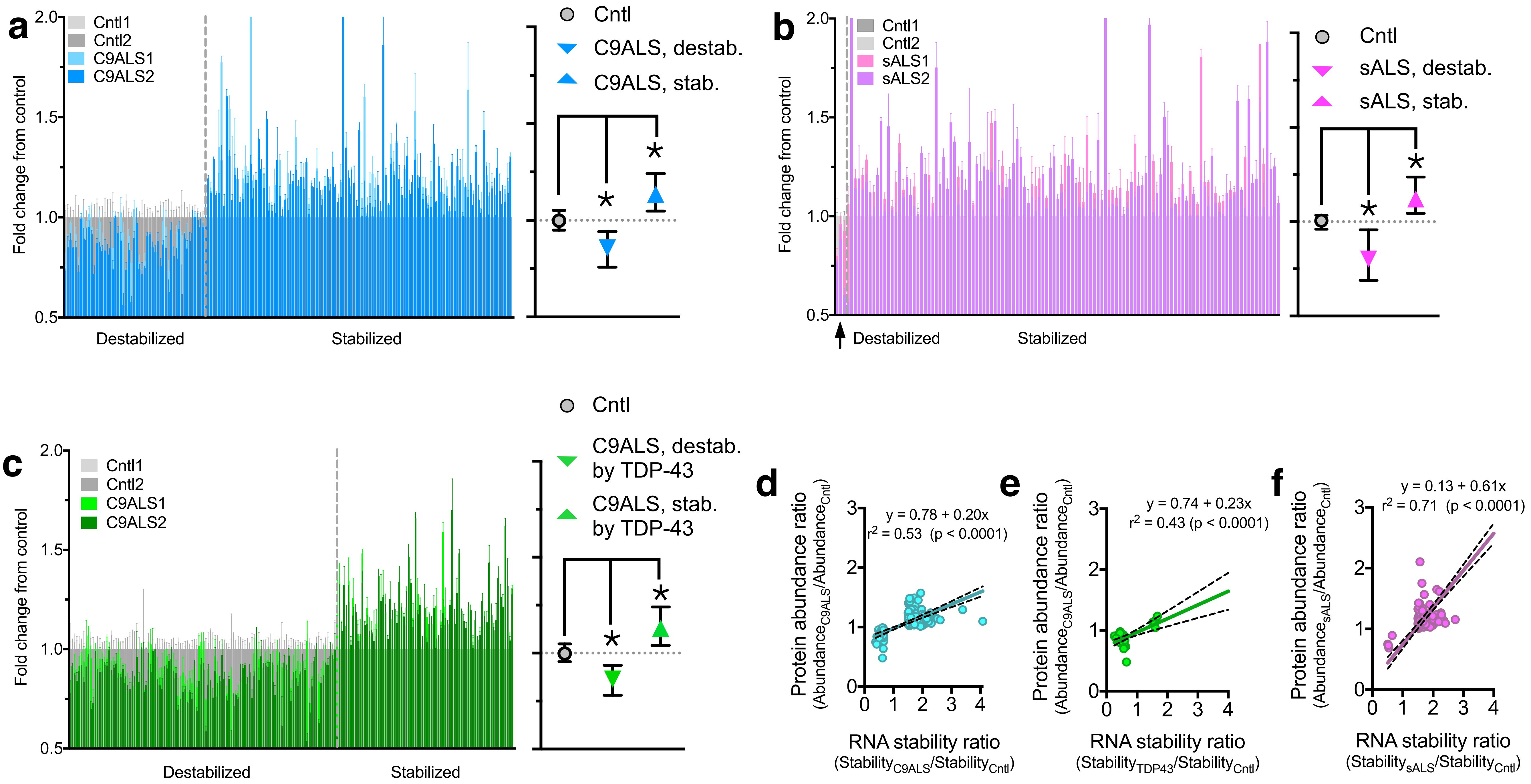

Supplementary figure 15: Concordant changes in RNA stability and protein abundance in ALS iPSCs. a 170 proteins demonstrated concordant changes in RNA stability and protein abundance in C9ALS iPSCs. **b** In sALS iPSCs, 121 proteins showed concordant changes in RNA stability and protein abundance. **c** 194 proteins exhibited concordant changes in RNA stability in TDP-43 overexpressing iPSCs and protein abundance in C9ALS iPSCs. In each case, the net mean changes in the abundance of these proteins differed significantly from controls (Cntl). **, p < 0.001 two-tailed t-test.**, p < 0.001 two-tailed t-test. **d-f** Linear correlation between RNA stability and protein abundance in C9ALS iPSCs (**d**), RNA stability in TDP43-overexpressing iPSCs and protein abundance in C9ALS iPSCs (**e**), and RNA stability and protein abundance in sALS iPSCs (**f**). Plots to the right of (**a-c**) show mean ± standard error, and p determined by one-way ANOVA with Dunnett's post-test. In (**d-f**), p determined by the extra sum-of-squares F test. Stab stabilized. Destab destabilized

Supplementary Figure 16

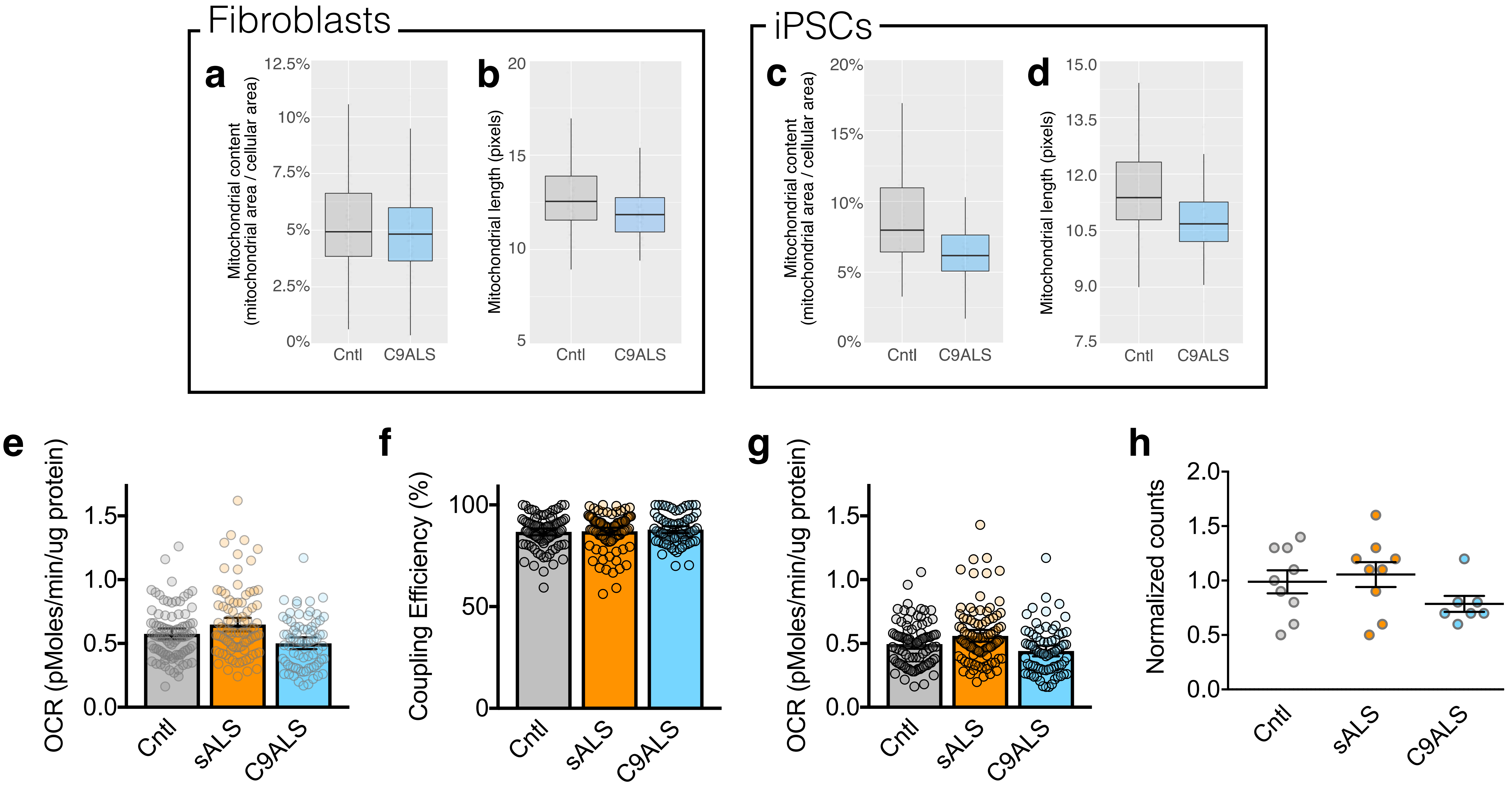

Supplementary figure 16: Oxidative phosphorylation and protein synthesis in ALS patient-derived cells. Mitochondrial content (percentage of cell area taken up by mitochondria) and length were no different in control and C9ALS fibroblasts (**a, b**) and iPSCs (**c, d**). Mitochondrial morphology was assessed in fibroblasts by transduction with mito-GFP, and by application of TMRE dye in iPSCs, as described in the Methods section. Plots in (**a**-**d**) show median (horizontal line), interquartile range (box) and maximum/minimum (vertical lines). Bioenergetics analyses demonstrated no significant change in mitochondrial oxygen consumption rate (OCR, **e**), coupling efficiency (**f**), or ATP-coupled respiration (**g**) in sALS and C9ALS fibroblasts, in comparison to controls (Cntl). n = 8 (Cntl), 8 (sALS) and 7 (C9ALS) lines per group, as described in Supplementary table 1. Plots in (**e**-**g**) show mean ± standard error. **h** Rates of protein translation, as measured by SUnSET, were not significantly different from one another in control, C9ALS and sALS fibroblasts. Plot shows normalized anti-puromycin counts (mean ± standard error) from 2 lines of each group, performed in triplicate

Supplementary Figure 17

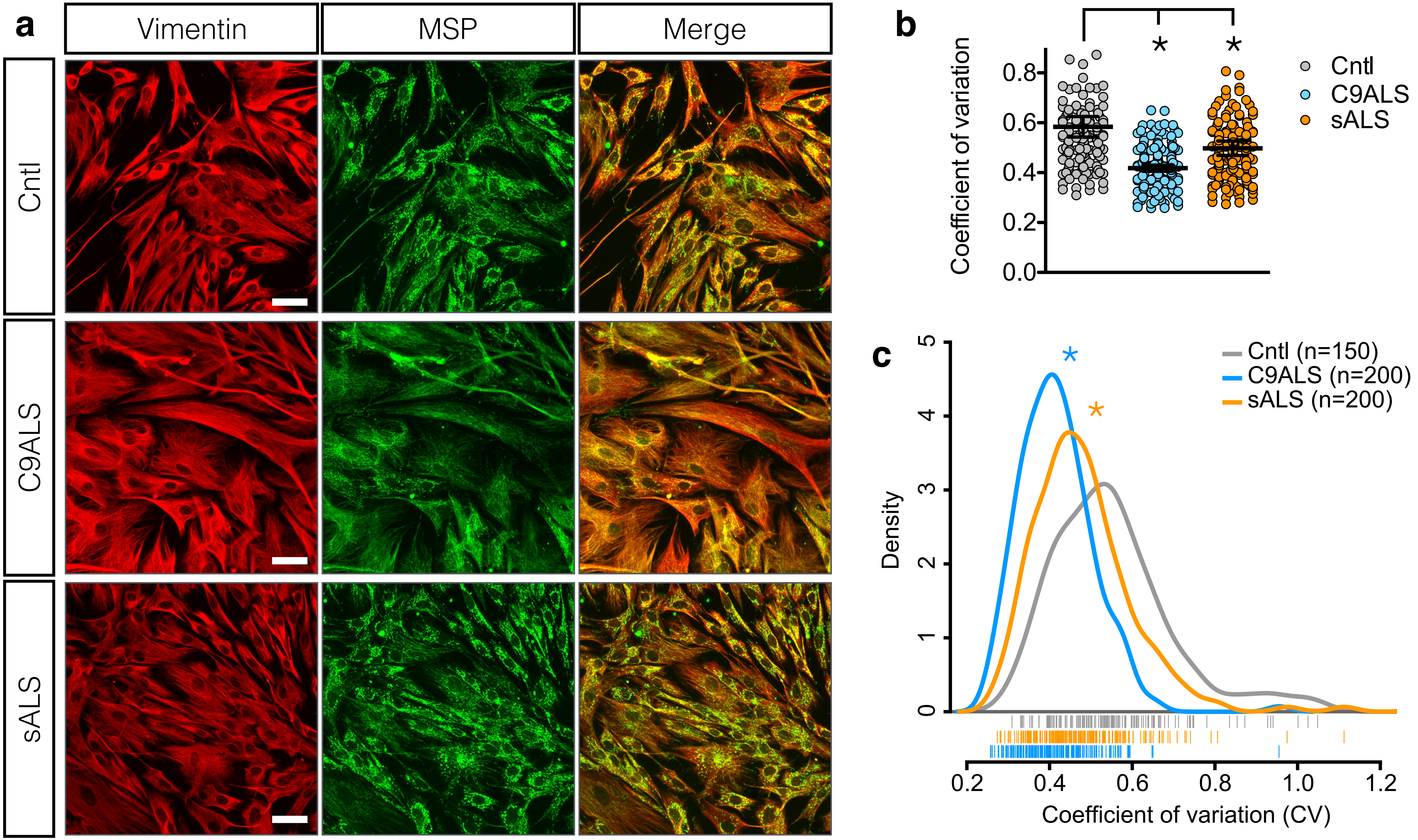

Supplementary figure 17: Mitochondrial morphology in fixed ALS fibroblasts. a Immunocytochemistry for vimentin and mitochondrial surface protein (MSP) in fibroblasts, demonstrating linear rather than punctate mitochondria in C9ALS cells. Scale bar, 50 μm. **b** The coefficient of variation of mitochondrial staining, a measure of granularity, was significantly reduced in C9ALS and sALS fibroblasts. *, $p < 0.0001$, one-way ANOVA with Dunnett's test. **c** Density plot of mitochondrial coefficient of variation, demonstrating significantly left-shifted distributions for C9ALS and sALS fibroblasts. *, $p < 0.0001$, Kolmogorov-Smirnov test. Observations collected from 150 cells (Cntl) or 200 cells (sALS, C9ALS) per genotype, combined from 12 separate lines of fibroblasts (4 Cntl, 4 sALS, and 4 C9ALS)

Supplementary Figure 18

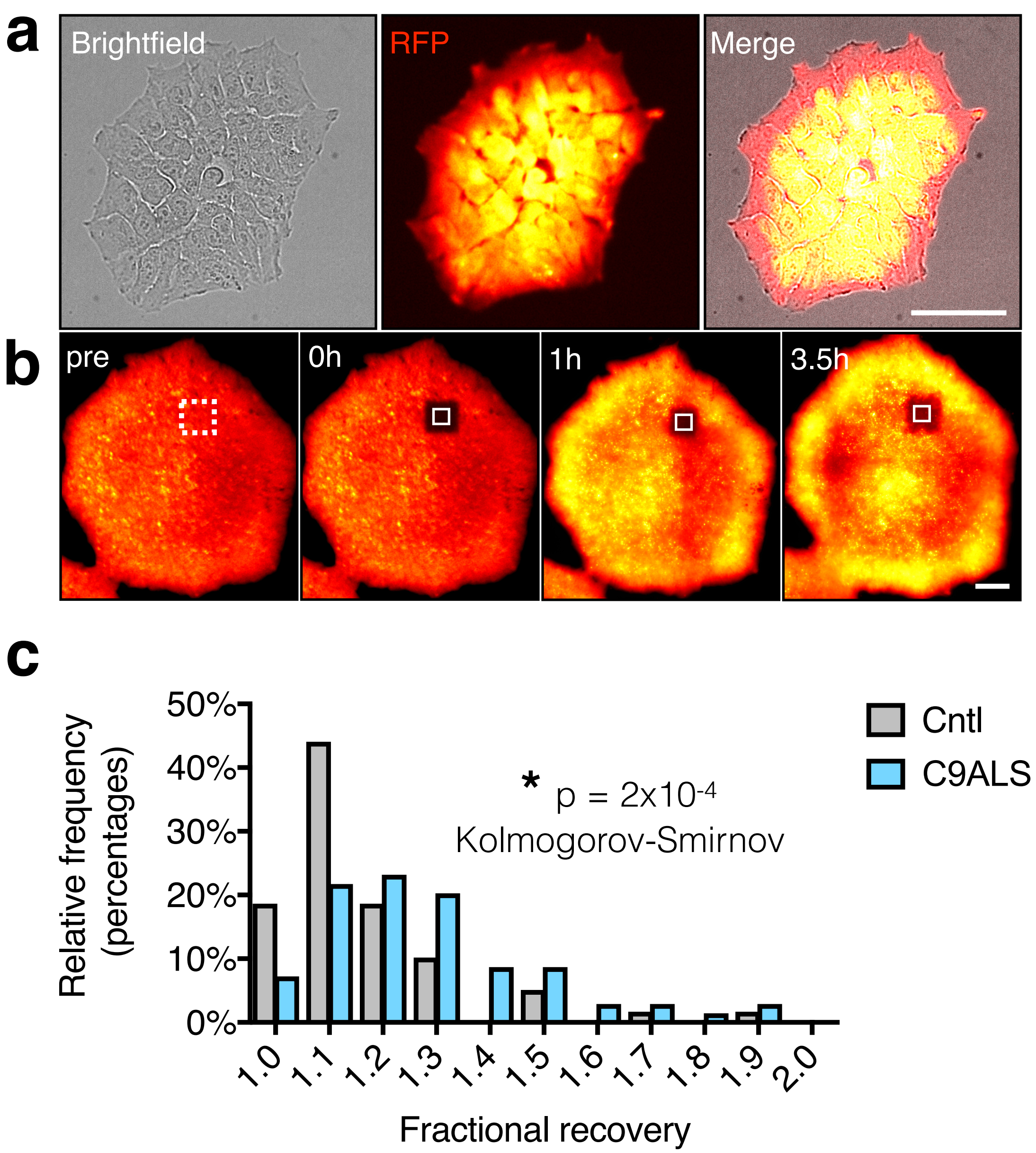

Supplementary figure 18: Recovery of fluorescence after photo-bleaching iPSCs expressing mCherry. a Baseline red fluorescence signal in iPSCs. **b** A small region within an iPSC colony was selected for photobleaching (dotted box). Fluorescence recovery measured within a smaller area, delimited by the solid white box. Scale bars, 100 μm. **c** Histogram of fractional recovery rates, demonstrating faster recovery in C9ALS iPSCs. Observations collected from 3 biological replicates of 2 iPSC lines per genotype
