# Supplementary material for: Abnormal RNA stability in amyotrophic lateral sclerosis
Source: Nat Commun. 2018 Jul 20;9:2845. doi: 10.1038/s41467-018-05049-z (PMC6054632; doi:10.1038/s41467-018-05049-z)
Supplement: Supplementary file 2 — Description of Additional Supplementary Files [file 41467_2018_5049_MOESM2_ESM.pdf]

Description of Additional Supplementary Files

File Name: Supplementary Data 1
Description: This file includes stability ratios measured by BruChase-seq for mapped transcripts from C9ALS and sALS fibroblasts, illustrated in Fig. 1.

File Name: Supplementary Data 2
Description: This file includes synthesis rates measured by Bru-seq for mapped transcripts in C9ALS and sALS fibroblasts, illustrated in Fig. 2.

File Name: Supplementary Data 3
Description: This file includes stability ratios measured by BruChase-seq for mapped transcripts from C9ALS and sALS iPSCs, as well as iPSCs transfected with TDP43-EGFP, illustrated in Figs. 3, 4 and 7, and Supplementary figs. 4, 10 and 15.

File Name: Supplementary Data 4
Description: This file includes synthesis rates measured by Bru-seq for mapped transcripts in C9ALS and sALS iPSCs, as well as iPSCs transfected with TDP43-EGFP, illustrated in Supplementary figs. 6 and 11.

File Name: Supplementary Data 5
Description: This file includes relative abundances of proteins in C9ALS and sALS iPSCs as detected by mass spectrometry, illustrated in Figs. 5-7, and Supplementary figs. 14 and 15.
